# Comprehensive network of miRNA-induced intergenic interactions and a biological role of its core in cancer

**DOI:** 10.1038/s41598-018-20215-5

**Published:** 2018-02-05

**Authors:** Vladimir V. Galatenko, Alexey V. Galatenko, Timur R. Samatov, Andrey A. Turchinovich, Maxim Yu. Shkurnikov, Julia A. Makarova, Alexander G. Tonevitsky

**Affiliations:** 1 Lomonosov Moscow State University, Leninskie Gory 1, 119991 Moscow, Russia; 2 SRC Bioclinicum, Ugreshskaya str. 2/85, 115088 Moscow, Russia; 3 Tauber Bioinformatics Research Center, University of Haifa, 199 Aba Khoushy Ave., Mount Carmel, 3498838 Haifa, Israel; 4 SciBerg e.Kfm, Birkenauer Str. 7, 68309 Mannheim, Germany; 5 P. Hertsen Moscow Oncology Research Institute, National Center of Medical Radiological Research, Second Botkinsky lane 3, 125284 Moscow, Russia; 6 Engelhardt Institute of Molecular Biology, Russian Academy of Sciences, Vavilova str. 32, 119991 Moscow, Russia; 7 Present Address: Evotec International GmbH, Marie-Curie Str. 7, 37079 Göttingen, Germany

## Abstract

MicroRNAs (miRNAs) are a family of short noncoding RNAs that posttranscriptionally regulate gene expression and play an important role in multiple cellular processes. A significant percentage of miRNAs are intragenic, which is often functionally related to their host genes playing either antagonistic or synergistic roles. In this study, we constructed and analyzed the entire network of intergenic interactions induced by intragenic miRNAs. We further focused on the core of this network, which was defined as a union of nontrivial strongly connected components, i.e., sets of nodes (genes) mutually connected via directed paths. Both the entire network and its core possessed statistically significant non-random properties. Specifically, genes forming the core had high expression levels and low expression variance. Furthermore, the network core did not split into separate components corresponding to individual signalling or metabolic pathways, but integrated genes involved in key cellular processes, including DNA replication, transcription, protein homeostasis and cell metabolism. We suggest that the network core, consisting of genes mutually regulated by their intragenic miRNAs, could coordinate adjacent pathways or homeostatic control circuits, serving as a horizontal inter-circuit link. Notably, expression patterns of these genes had an efficient prognostic potential for breast and colorectal cancer patients.

## Introduction

MicroRNAs (miRNAs) are a family of short (~22 nt) noncoding RNAs that posttranscriptionally regulate gene expression and play an important role in various cellular processes, including oncogenesis, epithelial–mesenchymal transition, regeneration, embryogenesis, and cellular differentiation^1–5^. Furthermore, miRNAs can function in coordination with various epigenetic regulators^6^ and transcription factors^7^. The miRNA concentration in a cell can rapidly change^8^, and therefore, miRNA expression is considered an element of early genetic response to external stimuli^9,10^. Finally, miRNAs also regulate cellular homeostasis by serving as nodes of signalling networks^11^.

A significant percentage of miRNAs are intragenic, i.e., located within intronic or exonic regions of coding genes (host genes)^12^. In humans, more than half of miRNAs are intragenic^13^. At the same time, the majority of intragenic miRNAs are located within introns^14^; specifically, humans intronic miRNAs constitute more than 85% of all intragenic miRNAs^13^. Moreover, intronic miRNAs are usually transcribed in the same direction as their host genes^14^. In humans, more than 80% of intronic miRNA genes have a sense orientation with respect to their host genes^13^. Therefore, most human intragenic miRNAs are co-transcribed with their host genes and subsequently released at the splicing stage^15,16^.

**Figure 1:**
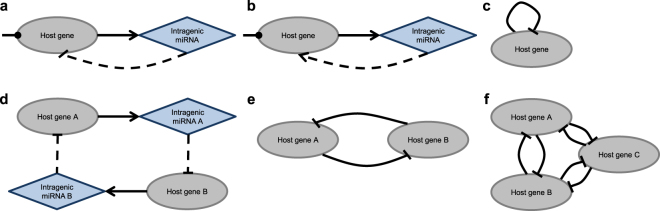
Regulatory network motifs involving intragenic miRNAs. (**а**) A self-regulatory negative feedback loop. (**b**) A self-regulatory positive feedback loop. (**c**) Representation of a self-regulatory feedback loop in the constructed network of intergenic interactions induced by intragenic miRNAs (note that loops are removed prior to the analysis of the network). (**d**) A pair of genes mutually targeting each other via their intragenic miRNAs. (**e**) Representation of miRNA-induced intergenic interactions shown in panel (d) in the constructed network. (**f**) A three-node sub-network in which each pair of nodes is mutually (bidirectionally) connected.

In addition to having overlapping genome locations, intragenic miRNAs and their host genes can be functionally connected; however, in studies demonstrating these links, the pairs of {host gene – intragenic miRNA} were analyzed independently from each other. Specifically, a number of theoretical and experimental studies have shown that the host genes can be the direct targets of their intragenic miRNAs. Targeting of a host gene by its intragenic miRNA was observed not only for exonic antisense miRNAs (which are complementary to a gene region but transcribed independently of their host genes)^17,18^ but also for multiple intronic and exonic sense miRNAs^19–23^. Furthermore, computational analysis demonstrated that depending on the parameters applied in the model, self-regulation of genes via their intragenic miRNAs can have various biological roles, including buffering of “expression noise”, conferring expression robustness, regulating the timing of responses to external signals, and adapting gene expression to persistent stimuli, thus providing responses conforming to Weber’s law^24^. Notably, a non-canonical mechanism of self-regulation of genes, where intragenic miRNA enhances transcription instead of mediating posttranscriptional repression, was also reported^25^. Co-transcription of a host gene and its targeting miRNA (followed by subsequent release of the miRNA precursor during splicing and its processing into a mature miRNA via a common well-described mechanism^16,26,27^) can be regarded as a negative (in most cases) or a positive (occasionally) feedback loop^28^ (Fig. 1a,b). This self-regulation of gene expression may be regarded as a specific case of regulatory network motifs that include both transcription factors and miRNAs^7,11,29,30^.

Other reported types of functional relations between host genes and their intragenic miRNAs include miRNA-targeting of genes whose products are downstream effectors of host gene products, genes antagonistic to a host gene, or genes belonging to the same pathway as a host gene^14,31–35^.

We hypothesized that regulatory network motifs involving intragenic miRNAs could simultaneously function in a cell as interconnected parts of the entire mechanism of gene expression regulation. Therefore, in the present study, we did not focus on individual regulatory motifs but, for the first time, constructed and analyzed the entire network of intergenic interactions induced by intragenic miRNAs. It is a gene oriented network which edges correspond to miRNAs and represent targeting of one gene by a miRNA hosted in the other gene. In particular, we identified condensed core of the constructed network. The core contained 21 or 12 genes (for all intragenic miRNAs or only intronic sense miRNAs, respectively) involved in key cellular processes, including DNA replication, transcription, protein homeostasis and cell metabolism. Intragenic miRNAs located in the core genes are likely to confer the robustness of the core by buffering internal and external noises^30,36–38^, fine-tune the expression of the core genes^39^ and, more generally, coordinate adjacent pathways or homeostatic control circuits by serving as a horizontal inter-circuit link. We further hypothesized that regulation of expression of the core genes mediated by their intragenic miRNAs could be important for normal cell functioning, and distortion of the expression patterns of these genes could have a significant diagnostic potential. As proof of concept, we identified gene expression signatures consisting solely of core genes for highly efficient recurrence prognosis of breast and colorectal cancer. Remarkably, these expression signatures were as efficient as ones identified from a genome-wide transcriptome analysis^40,41^.

## Results

### The network design

The construction and analysis of the network of intergenic interactions induced by intragenic miRNAs were performed on the levels of (1) all intragenic miRNAs and (2) only intronic sense miRNAs. For network construction we used only validated targets of miRNAs. Therefore, we focused on humans since human databases of validated miRNA targets are currently the most comprehensive.

The designed network is gene oriented and has the following structure. Its nodes are genes, and nodes (genes) *A* and *B* are connected by a directed edge (*A* –┤ *B*) if gene *A* is a host for an intragenic miRNA that targets gene *B*. We use bar-headed arrows to represent edges as miRNAs generally suppress their targets.

Specifically, a trivial cycle (loop) *A* –┤ *A* (Fig. 1c) in this network represents self-regulation of gene *A* by its intragenic miRNA. In addition to the loops, the resulting network contains nontrivial cycles, including pairs of mutually connected genes (Fig. 1d,e). To the best of our knowledge, this type of regulatory network pattern was not previously reported and analyzed for miRNA-induced intergenic interactions. At the same time, there are no triples in which each pair of genes is mutually (bidirectionally) connected. In other words, the network does not contain a sub-network depicted in Fig. 1f.

However, if not confined to one-edge paths (i.e., to direct miRNA-induced interactions) and considering longer paths with intermediate nodes, two relatively small components comprising nodes mutually connected by directed paths can be revealed. In other words, the network contains exactly two nontrivial (i.e., containing at least two nodes) strongly connected components. These components can be considered analogues of the host genes that are self-regulated by their intragenic miRNAs. Each node of a component is a host gene whose intragenic miRNA targets the members of the same component (although a direct miRNA-induced interaction exists not for all pairs of genes). And vice versa, each node is a gene targeted by a miRNA hosted in another gene from this component.

The union of the nontrivial strongly connected components will be further referred to as a “network core”. Section *Network core: a simple example* of Supplementary Information illustrates the definition of the network core.

### Quantitative characteristics of networks

A full network (F-network) of miRNA-induced intergenic interactions in humans was designed based on the lists of experimentally validated miRNA targets. A target was considered validated if it was present simultaneously in two databases of validated miRNA targets — DIANA-TarBase v7.0^42^ and miRTarBase (Release 6.1)^43^.

The miRNA–target interactions were validated in total for 842 mature miRNAs, 445 of which were intragenic. Furthermore, these 445 mature miRNAs originated from 389 pre-miRNAs located in 305 different host genes and targeted 8,416 genes. The targeted genes included 176 out of 305 host genes. Consequently, the F-network contained 176 nodes with both in- and out-edges, 129 nodes with only out-edges and 8,240 nodes with only in-edges (see Supplementary Fig. 1 as an illustration): 8,545 non-isolated nodes in total. The number of edges in the F-network (after removing loops and merging multiple edges) was 19,081. These numbers are summarized in Table 1.

**Table 1:** Quantitative characteristics of the F-network and IS-network.

	**TotalN**	**BothDirN**	**OutN**	**InN**	**TotalE**	**Mat-mi**	**Pre-mi**	**HostGenes**
F-network	8,545	176	129	8,240	19,081	445	389	305
IS-network	8,307	140	106	8,061	16,913	364	277	246

TotalN – total number of non-isolated nodes. BothDirN – number of nodes with both in- and out-edges. OutN – number of nodes with out-edges but no in-edges. InN – number of nodes with in-edges but no out-edges. TotalE – total number of edges after removing loops and merging multiple edges. Mat-mi – number of mature miRNAs that induce intergenic interactions represented as network edges. Pre-mi – number of pre-miRNAs for these mature miRNAs. HostGenes – number of genes hosting these pre-miRNAs. Clearly, TotalN = BothDirN + OutN + InN, HostGenes = BothDirN + OutN.

Along with the F-network, which was based on all intragenic miRNAs, we constructed an IS-network based solely on intronic sense miRNAs. In the IS-network, the number of different nodes and edges was evidently lower compared to that of the F-network; however, this difference was minor since intronic sense miRNAs constitute the majority of all intragenic miRNAs (Table 1).

Formal description of the constructed networks through the listing of their edges is presented in Supplementary Data (worksheets *F-network formal descr*. and *IS-network formal descr*.).

### Nodes with the highest in-degree

The analysis of lists of nodes with the highest in-degree (i.e., lists of genes having the highest regulation by intragenic miRNAs) revealed a moderate enrichment for several major functional categories. Specifically, for both networks, the enrichment was evident for such categories as cell cycle, p53 signalling pathway, oncogenic pathway, focal adhesion, apoptosis, and transcriptional and posttranscriptional regulation of gene expression (Table 2). Additional data are presented in Supplementary Information (section *The enrichment analysis of lists of nodes with the highest in-degree*). Histograms of in-degree for the F-network and the IS-network are presented in Supplementary Data (worksheets *F-network in-degree hist*. and *IS-network in-degree hist*).

**Table 2:** Major functional categories overrepresented in lists of genes with the highest in-degree.

Term	F-network adj. p-val	IS-network adj. p-val
Cell cycle	1.09 × 10^−5^	1.16 × 10^−5^
p53 signalling pathway	3.11 × 10^−5^	3.62 × 10^−5^
Pathways in cancer	2.15 × 10^−5^	4.24 × 10^−4^
Focal adhesion	6.54 × 10^−4^	5.25 × 10^−4^
Apoptosis	1.17 × 10^−3^	1.21 × 10^−3^
Posttranscriptional regulation of gene expression	3.32 × 10^−5^	1.63 × 10^−3^
Transcription regulation	4.12 × 10^−3^	1.19 × 10^−2^

F-network adj. p-val – Benjamini-corrected p-value for the F-network list reported by DAVID; IS-network adj. p-val – Benjamini-corrected p-value for the IS-network list reported by DAVID.

### Overrepresented three-node network motifs

**Figure 2:**
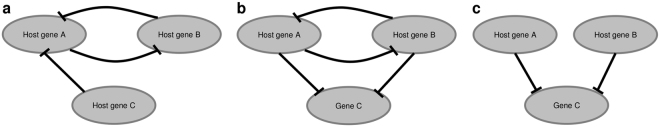
Three-node network motifs overrepresented in the F-network and IS-network in comparison with random graphs (**a**) generated using a model with a fixed set of out-degrees of vertices; (**b**) generated with the Erdős–Rényi model; (**c**) generated using any of these two models.

Both F- and IS-networks had properties different from those of random graphs with the same number of vertices and edges. Notably, the number of occurrences of the three-node network motif presented in Fig. 2a (41 for the F-network and 18 for the IS-network) was significantly higher than the expected number for this motif in a random graph. For random graphs generated using a model with a fixed set of out-degrees of vertices (which is equivalent to the randomization of lists of miRNA targets preserving sizes of the lists), p-values did not exceed 10^−4^ for the F-network and 0.017 for the IS-network (see Supplementary Table 5 – sub-network type 6). For random graphs generated using the Erdős–Rényi model^44^, the p-value was 2.6 × 10^−3^ for the F-network, but for the IS-network the statistical significance was lower, with a p-value of 0.1. However, a clear difference between the constructed networks and random graphs generated with the Erdős–Rényi model was revealed by the analysis of other three-node motifs. E.g., an evident overrepresentation in comparison with Erdős–Rényi– random graphs was observed for a motif shown in Fig. 2b (p-value < 10^−4^ for both the F-network and the IS-network; see Supplementary Table 5 – sub-network type 14).

Furthermore, a three-node network motif presented in Fig. 2c was also overrepresented in the constructed networks in comparison with random graphs. The number of its occurrence in the F-network and in the IS-network was 30757 and 22913, respectively, which resulted in p-values < 10^−4^ for both the Erdős–Rényi model and the model with a fixed set of out-degrees (see Supplementary Table 5 – sub-network type 3).

Aggregate information on the number of non-equivalent (non-isomorphic) three-node sub-networks in the constructed networks and in random graphs is presented in Supplementary Information (section *Three-node sub-networks*). Histograms supporting the specified p-values are also presented in Supplementary Information (section *Histograms supporting p-values*).

### The network core

**Figure 3:**
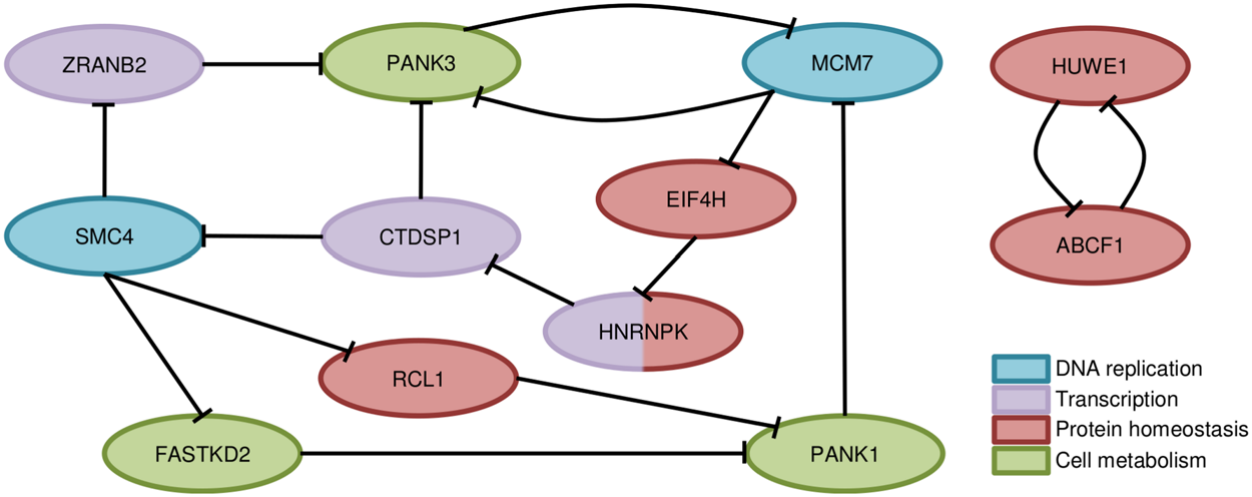
The core of the IS-network. The nodes are genes, and nodes (genes) *A* and *B* are connected by a directed edge (*A* –┤ *B*) if gene *A* is a host for an intronic sense miRNA that targets gene *B*.

For the IS-network, the core consisted of two strongly connected components that included 10 and 2 genes (Fig. 3; Supplementary Data – worksheet *IS-network core formal descr*.). The core of the F-network contained the IS-network core as a subgraph and also consisted of two components, with 18 and 3 genes (see Supplementary Fig. 6 and Supplementary Data – worksheet *IS-network core formal descr*.).

Similarly to the three-node network motifs discussed above, network core size clearly differentiated both the F-network and the IS-network from random graphs generated using the Erdős–Rényi model (for random graphs, the size of the core was substantially higher, p-value < 10^−4^). Applying the model with a fixed set of out-degrees of vertices resulted in the reduction of the core size in random graphs: the core size for the F- and IS-networks was still smaller than a median core size for random graphs, but statistical significance was violated (p-value was close to 0.17 for both the F- and IS-networks). Histograms supporting the specified p-values are presented in Supplementary Information (subsection *Histograms supporting p-values*).

The network core comprised genes that are essential for key cellular processes. For example, *MCM7* and *SMC4* are indispensable for DNA replication: *MCM7* promotes unwinding of dsDNA in the replication fork^45^ and *SMC4* is involved in condensation of mitotic chromosomes^46^. Furthermore the *CTDSP1* and *ZRANB2* genes are important transcriptional regulators responsible for gene silencing and alternative splicing, respectively^47,48^. Four other genes from the IS-network core are involved in translation control and maintenance of protein homeostasis. *EIF4H* and *ABCF1* are both involved in translation initiation^49,50^. *RCL1* encodes a nuclease that cleaves pre-rRNA at the A2 site, thus separating rRNA destined for small and large ribosomal subunits^51^. The product of the *HUWE1* gene is a E3 ubiquitin-protein ligase that mediates ubiquitination and subsequent proteasomal degradation of target proteins, including the anti-apoptotic protein Mcl1, p53, c-Myc and core histones^52^. The protein encoded by *HNRNPK* is a key regulator of transcription and translation^53^. The *PANK1*, *PANK3* and *FASTKD2* genes are essential for basic cellular metabolism. *PANK1* and *PANK3* genes encode isoforms of pantothenate kinase, which catalyzes the first rate-limiting step of panthotenate biosynthesis. At the same time, pantothenate is the precursor of coenzyme A — a key cellular metabolite that serves as a cofactor for multiple enzymes and is involved in various processes, including fatty and amino acid biosynthesis, cell signalling and regulation of gene expression^54^. Finally, the *FASTKD2* protein is localized in the mitochondria, and its knockdown leads to the impairment of cellular respiration^55^.

To summarize, the network core connects the genes involved in basic cellular processes: DNA replication, transcription, protein homeostasis and underlying metabolic activity.

### High expression levels and low expression variance of the core genes

The analysis of genome-wide transcriptome profiles of 675 cell lines^56^ revealed that genes belonging to the cores of the IS-network and F-network have generally high expression levels. More specifically, after ranking the transcripts present in the dataset (ca. 26 thousand transcripts) by the median expression level, 10 out of 12 core genes from the IS-network were in the upper quarter, while two remaining genes were still in the upper half (binomial test p-value < 4 × 10^−5^). Similar ranking by the third (higher) or the first (lower) quartile led to similar results. Specifically, the distribution among quarters was exactly the same after ranking by the third quartile, while after the first quartile ranking, 11 out of 12 genes were located in the upper quarter, and the remaining gene was still found in the upper half. For the core of the F-network, statistically significant enrichment of highly expressed transcripts was even more remarkable. For example, after ranking by the median expression levels, 16 out of 21 genes comprising the core of the F-network resided in the upper quarter, and the remaining 5 genes were still in the upper half, with the binomial test p-value less than 10^−5^.

At the same time, genes from the core were not the most highly expressed ones: after ranking of transcripts by a median expression level, as well as by the first quartile, only one gene from the core (*HNRNPK*) appeared in the top 1%. After ranking by the third quartile, *HNRNPK* moved down (but remained in the top 2% zone), and no genes from the core were present in the top 1%.

Along with high expression, genes from the core have generally low variance in expression levels. Even after excluding low expression transcripts from the analysis and retaining the upper half of the transcripts (with respect to the ranking by the first quartile of expression levels), most genes from the core were in the half with a lower variance — 10 genes for the core of the IS-network (binomial test p-value 0.019) and 16 genes for the core of the F-network (binomial test p-value 0.013). Moreover, 8 genes were in the quarter of transcripts with the lowest variance for the core of the IS-network (binomial test p-value 0.0028), and for the core of the F-network, the number of such genes was 12 (binomial test p-value 0.0017).

Boxplots illustrating the described data are presented in Supplementary Information (Supplementary Figs 7, 8). Details of the analysis, including measures used to quantify expression levels and expression variance, are described in *Methods* section (subsection *Analysis of expression levels*).

Further analysis of expression levels of genes comprising the core in tissue specimens from 298 breast cancer patients and 519 colorectal patients confirmed these results. Details are presented in Supplementary Information (section *Expression levels of core genes in tissue specimens*).

Therefore, our data strongly indicate that the genes comprising the network core of intragenic miRNA-induced gene-gene interactions can be characterized as generally having higher expression levels and lower variance compared to the median values.

### The prognostic power of genes belonging to the core

Analysis of expression pattern of genes within the core led to clear conclusions about a cell and even a whole organism. More specifically, highly reliable prognostic gene signatures were derived for breast and colorectal cancer using only expression patterns of the genes from the network core.

These prognostic gene signatures were constructed separately for breast cancer and colorectal cancer using an SVM-based exhaustive search strategy described previously^40^. Microarray data utilized for this construction are specified in *Methods* section (subsection *Construction of prognostic gene signatures*). Both for breast cancer and for colorectal cancer these data included one training, two filtration and one validation (testing) dataset. For breast cancer the datasets reported expression levels for 17 genes from the core of the F-network, including 10 genes from the core of the IS-network; for colorectal cancer the datasets reported expression levels of all genes comprising the core of the F-network. Log-scaled expression levels were used.

For each gene its mean expression level and standard deviation of expression level were estimated based on the training dataset, and expressions in all datasets were (0, 1)-scaled by subtracting these means and dividing by these deviations.

Then for every studied subset of genes from the core of the F-network, ranging from individual genes to the whole core of the F-network, a binary classifier was constructed using Support Vector Machine^57^ (SVM) with the linear kernel and class weights inversely proportional to the number of patients in each class. This construction utilized only the training dataset. For triples of genes the classifier had a form

$$R({e}_{{\rm{1}}},{e}_{{\rm{2}}},{e}_{{\rm{3}}})={w}_{1}\,{e}_{{\rm{1}}}+{w}_{{\rm{2}}}\,{e}_{{\rm{2}}}+{w}_{{\rm{3}}}\,{e}_{{\rm{3}}}\,-\,\rho ,$$

where *e*_1_, *e*_2_, *e*_3_ were (0, 1)-scaled expressions (with scaling parameters derived from the training dataset), and weights *w*_1_, *w*_2_, *w*_3_ as well as *ρ* were computed using a standard SVM construction algorithm. Taking expression levels *e*_1_, *e*_2_, *e*_3_ associated with a patient, this classifier attributed the patient to the high risk group if *R* (*e*_1_, *e*_2_, *e*_3_) was nonnegative, and to a low-risk group otherwise. For sets with another number of genes the difference in the classifier forms was only in the number of terms in a linear combination.

If the resulting classifier did not pass thresholds on sensitivity and specificity for the training dataset (the utilized values are specified in *Methods* section, subsection *Construction of prognostic gene signatures*), it was excluded from the further analysis. Otherwise, in order to avoid overfitting, this classifier was additionally applied to each of the filtration datasets. If sensitivity or specificity for at least one filtration dataset was lower than a filtration threshold, the classifier was also filtered out. For classifiers that passed the filtration their prognostic characteristics were estimated using the validation dataset. Samples from the validation dataset were utilized neither for classifier construction, nor for filtration.

**Figure 4:**
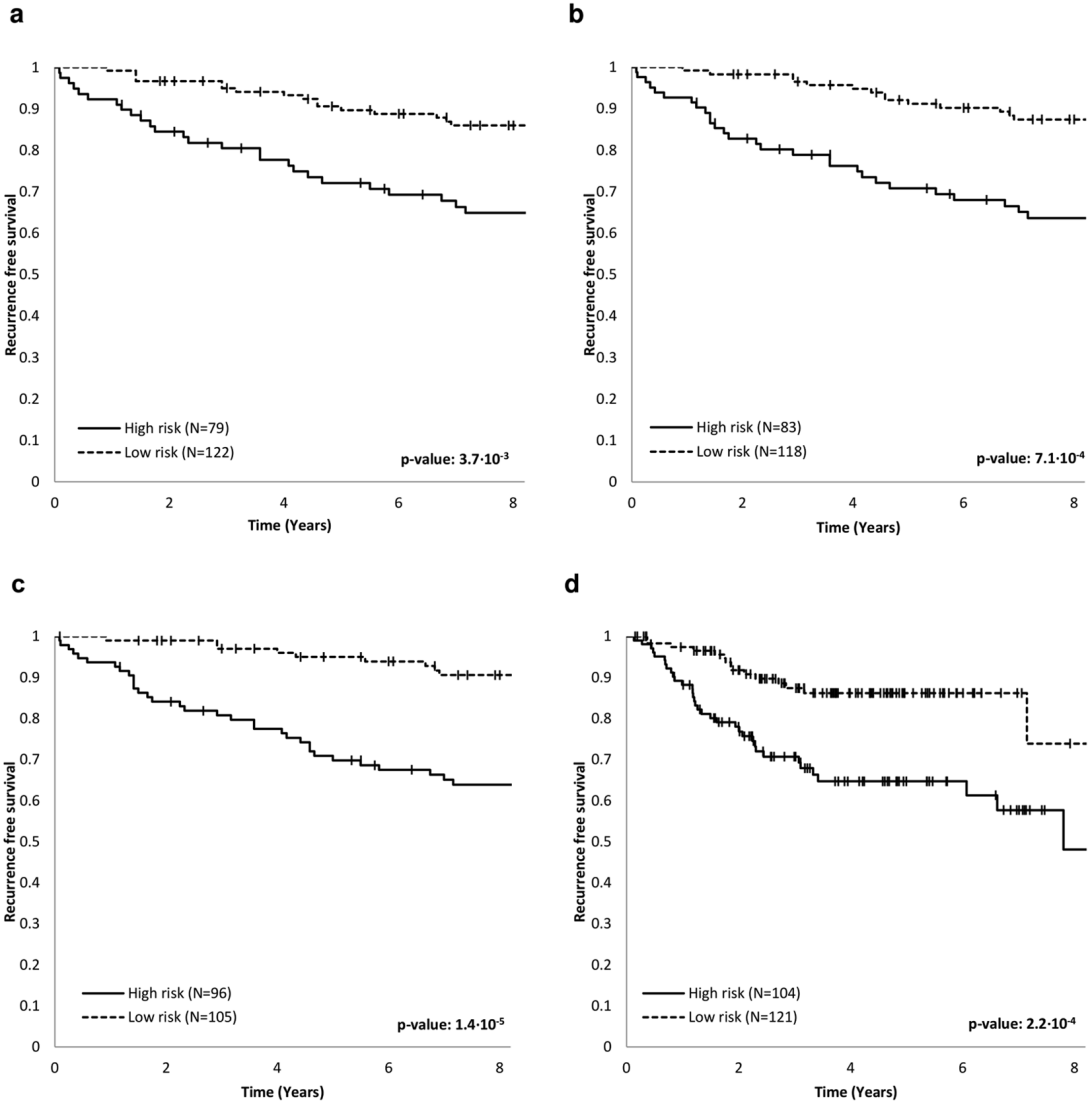
Kaplan-Meier curves for prognostic three-gene signatures. Log-rank p-values are reported. (**a**) Breast cancer, genes *HNRNPK, PANK3, SMC4*. (**b**) Breast cancer, genes *MCM7, PANK3, SMC4*. (**c**) Breast cancer, genes *MAP2K4, PANK3, SMC4*. (**d**) Colorectal cancer, genes *FASTKD2, PANK1, HUWE1*.

Limiting the analysis to the triples comprising the genes from the core of the IS-network, we identified *HNRNPK*, *PANK3* and *SMC4* gene triple as having the highest prognostic power for estrogen receptor-positive (ER-positive) breast cancer (with respect to the training and filtration datasets – see *Methods*, subsection *Construction of prognostic gene signatures*). Furthermore, on the validation dataset this triple provided prediction of breast cancer recurrence with a sensitivity and specificity of 63.6% and 66.4%, respectively. The AUC value for the validation dataset was 0.662, while its mean value for the training and filtration datasets was equal to 0.708. The Kaplan-Meier curves for the validation dataset are presented in Fig. 4a.

The genes *HNRNPK*, *PANK3* and *SMC4* are not involved in one cellular process but contribute to different pathways related to DNA replication (*SMC4*), regulation of transcription and translation (*HNRNPK*) and cellular metabolism (*PANK3*). Notably, *SMC4* and *PANK3* genes had the highest degree (i.e., total number of in- and out-edges for a node) in the subgraph of the IS-network induced by its core (Fig. 3). Interestingly, the substitution of *HNRNPK* for *MCM7* (the remaining gene with degree 4) yielded a three-gene signature with a similar prognostic power for the training and filtration datasets (mean AUC value 0.693) and even higher characteristics for the validation dataset (sensitivity 69.7%, specificity 66.4%, AUC 0.670; Fig. 4b).

Extension of the *SMC4*-*PANK3* pair to the triple with *MAP2K4*, which belongs to the core of the F-network, slightly enhanced the mean AUC value for the training and filtration datasets and increased the sensitivity, specificity and AUC value for the validation dataset to 84.8%, 60.2% and 0.728, respectively (Fig. 4c).

Therefore, the analysis limited to the core of the network of intragenic miRNA-induced gene-gene interactions allowed construction of three-gene signatures for the prognosis of ER-positive breast cancer recurrence with sensitivity and specificity comparable to the most reliable three-gene signatures identified by extensive genome-wide analysis^40^ as well as the reliability of multigene commercial prognostic signatures, such as OncotypeDX^58,59^ and MammaPrint^59,60^.

For colorectal cancer, an exhaustive search revealed the *FASTKD2*, *PANK1*, and *HUWE1* gene triple from the core to have the highest prognostic power (with respect to the training and filtration datasets). All these genes belong to the core of the IS-network, but this triple was also optimal for the core of the F-network. The sensitivity and specificity of this triple in predicting the recurrence for colorectal cancer on the validation dataset were 68.3% and 60.0%, respectively, while the AUC value was 0.659 (Fig. 4d). These values of sensitivity and specificity were similar to the ones for the OncotypeDX for Colon Cancer^41^, which was designed based on a large-scale transcriptome analysis (expression levels were measured for 761 preselected candidate genes)^41,61^.

It is worth mentioning that the prognostic power of the above described triples of genes from the network core was provided by the totality of all genes in a triple. For gene pairs (and, obviously, for individual genes), the prognostic power was much lower. Moreover, none of the pairs of genes from the core passed the filtration.

Individual genes in each triple had different weights *w* (which characterize prognostic potential) in the resulting classifier value. The *SMC4* gene had the highest weight in all the above described triples for breast cancer, and increased expression of this gene (in the case of unaltered expression levels of the other two genes in the triple) was associated with a higher risk of recurrence. These data are consistent with emerging reports on the oncogenic potential of the *SMC4* gene^62,63^. Similarly, elevated expression of the *MCM7* gene, whose oncogenic potential has been widely discussed^64–66^, was also associated with a higher risk of cancer recurrence. For *PANK3*, *HNRNPK* and *MAP2K4* genes, this correlation was opposite. Two of these genes (*HNRNPK* and *MAP2K4*) were previously identified as tumor suppressors^67–69^; however, a pro metastatic-role of these genes was also reported^70,71^. To the best of our knowledge, the role of *PANK3* in oncogenesis has not yet been described.

In the case of the *FASTKD2-PANK1-HUWE1* triple, a higher risk of cancer recurrence was associated with increased expression of the *HUWE1* gene and lower expression levels of *FASTKD2* and *PANK1*. The *FASTKD2* gene, which has the greatest weight in the classifier for this triple, has been previously shown to promote apoptosis of cancer cells^72^. Furthermore, both oncogenic and tumor suppression roles were previously reported for the *HUWE1* gene^73–76^. In contrast, as for the *PANK3* gene, the role of the *PANK1* gene in the oncogenesis remains unknown.

Interestingly, larger gene sets (e.g., quadruples) did not provide a substantial increase in prognostic power. Moreover, the reliability of cancer prognosis based on the set consisting of all genes in the core was even lower in comparison with the most reliable triples due to overfitting of the classifier.

Weights of genes in the above classifiers and additional details of triples filtration are presented in Supplementary Information (section *Prognostic gene triples*).

### A connection between the IS-network core and the Myc protein

Notably, a striking functional connection between the core of the IS-network and the Myc protein has been found. The transcription factor Myc is known to have a high number of different targets and has a pronounced oncogenic potential^77–79^. Analysis of the published data revealed that 9 out of 12 genes comprising the core were either regulated by Myc directly or shown to conversely interact with Myc at the protein level, regulating its activity. See Supplementary Information for details (section *Interplay of the IS-network core and Myc*).

## Discussion

Despite the fact that a typical miRNA-target interaction mediates only a subtle reduction in protein levels^36,80^, miRNAs play an important physiological role by conferring robustness of biological processes^36,37^, fine-tuning essential cellular pathways, controlling signal transduction^11,39,81,82^ and maintaining metabolic homeostasis^83,84^. In particular, one report showed that aberrant levels of miR-103 and miR-107, intronic sense miRNAs located in the pantothenate kinase genes *PANK3* and *PANK1*, respectively, induced impaired glucose homeostasis^85^.

Over the last years, miRNAs have been shown to have significant impact in cancer, playing a role in each step of cancer biogenesis and progression^86,87^. Numerous networks involved in different cancer types and comprising miRNAs as well as the target genes have been identified^88,89^. The reported networks proved that miRNAs can generate connected graphs and revealed cancer type-specific patterns, pointing out to diagnostic, prognostic and therapeutic potential of miRNAs and miRNA-based networks^90^.

Intragenic miRNAs constitute a significant percentage of total miRNAs^12,13^ and are often functionally related with their host genes^14^, playing either antagonistic or synergistic roles^31,33,35^. In the present study, instead of focusing on individual regulatory network motifs or disease-specific networks involving miRNAs, we analyzed the entire network of intergenic interactions induced by either intronic sense miRNAs or all intragenic miRNAs. Furthermore, in these networks, we focused on the core, which has been defined as a union of nontrivial strongly connected components, i.e., sets of genes mutually connected by directed paths.

Generally, the genes belonging to the core had high expression levels and low expression variance. The latter is consistent with the concept of the miRNome as an instrument for conferring robustness of gene expression^30,36,37^ and maintaining cellular homeostasis^83,84^. Interestingly, the core could not be split into the components corresponding to individual signalling or metabolic pathways, but it integrated genes involved in the key cellular processes, including DNA replication, transcription, protein homeostasis and cell metabolism. Thus, interactions between genes from the core mediated by intragenic miRNAs could be involved not only in independent pathways or homeostatic control circuits^91^ but also in a horizontal regulation that subtly coordinates adjacent circuits.

Remarkably, the expression pattern of genes forming the core was shown to be an efficient prognostic marker in breast and colorectal cancer patients. While individual genes, as well as gene pairs, had relatively low prognostic power, a number of triples of genes from the network core provided a prognostic reliability comparable to that of the gene expression signatures identified by large-scale transcriptome analyses, including common commercial prognostic gene signatures. Furthermore, the higher risk of cancer recurrence was associated with differently directed changes in expression of genes for all identified triples, i.e., higher imbalance in the expression patterns of core genes.

The impact of expression changes of individual genes on the values of constructed prognostic classifiers was consistent with the previous reports on oncogenic or tumor suppressive roles of these genes. However, in the prognostic classifier for breast cancer, the weight of the well-studied *MCM7* oncogene^64–66^ was essentially lower than the weight of the *SMC4* gene with less reported oncogenic potential^62,63^. Similarly, there is limited evidence for the tumor suppressor potential of the *FASTK2* gene, which had the highest weight in the prognostic three-gene expression signature for colorectal cancer^72^.

To the best of our knowledge, the roles of *PANK1* and *PANK3* in oncogenesis have not been yet confirmed. However, transcription of *PANK1* has been previously reported to be directly targeted by p53^92^.

To summarize, we constructed a network of intergenic interactions induced by intragenic miRNAs and identified its core, which consists of genes involved in the key cellular processes. Both the network and its core possessed statistically significant non-random properties. Specifically, genes forming the core generally had high expression levels and low expression variance. Importantly, our data indicate that the expression pattern of these genes could be used for a reliable prognosis of recurrence for breast and colorectal cancer patients. Finally, we hypothesized that miRNA-induced intergenic interactions represented by directed edges in the network core could orchestrate subtle coordination of adjacent pathways and/or homeostatic control circuits by serving as horizontal inter-circuit regulatory links.

## Methods

### Network construction and analysis

The list of identifiers for intragenic pre-miRNAs and their respective host genes was downloaded from the miRIAD intragenic microRNA database^13^. The information for the classification of intragenic miRNAs into intronic and exonic, as well as their sorting into sense and antisense miRNAs, was also derived from this database. The identifiers of mature miRNAs corresponding to pre-miRNAs were obtained from the miRBase database^93,94^ (Release 21). The lists of validated targets of mature miRNAs were downloaded from the DIANA-TarBase v7.0^42^ and miRTarBase (Release 6.1)^43^ databases. An mRNA target was considered validated if both databases confirmed the targeting by a corresponding miRNA.

The analysis of the resulting networks was performed using the igraph library^95^. Multiple edges and loops, as well as isolated nodes, were removed prior to the analysis. This graph simplification used igraph R-package command *simple*.

The *network core* was defined as the union of nontrivial (i.e., containing at least two nodes) strongly connected components. These components were found using igraph command *components* with parameter *mode* = *“strong”*.

The comparison of properties of the constructed networks with the properties of random graphs was performed using the Erdős–Rényi model^44^ and a model based on fixed out-degrees. For both models, the number of vertices and edges coincided with the number of nodes and edges in the analyzed networks of miRNA-induced intergenic interactions. For the Erdős–Rényi model, the presence of an edge with given starting and ending vertices was equiprobable for all ordered pairs of vertices. For the second model, a number of outgoing edges *N*_*g*_ was fixed for each vertex *g*, and all possible sets of ending vertices for these edges (i.e., sets consisting of exactly *N*_*g*_ vertices and not containing *g*) were equiprobable.

Random graph generation under the Erdős–Rényi model was performed using igraph R-package command *erdos.renyi.game* with parameters *type* = *“gnm”, directed* = *TRUE, loops* = *FALSE*.

Generation of random graphs under the second model was performed as follows. Let |*V|* be the number of nodes (genes) in the analyzed networks of miRNA-induced intergenic interactions, and let (*N*_1_, *N*_2_, …, *N*_|*V*|_) be a vector of out-degrees. We obtained this vector using *igraph* command *degree* with parameter *mode* = *“out”*. Random graph generation started from a graph with |*V*| nodes {1, 2, …, |*V*|} and no edges. Then for each *k* from 1 to |*V*| a random permutation on {1, 2, …, |*V*| − 1} was generated using the Fisher-Yates shuffle^96^, first *N*_*k*_ elements of the generated permutation were selected, and for each selected element *l* a directed edge from node *k* to node *l* (if *l* < *k*) or to node (*l* + 1) (if *l* ≥ *k*) was added to the graph.

Distribution of properties of random graphs was assessed using the standard Monte Carlo approach after generating 10,000 graphs for each model.

The enrichment analysis was performed using DAVID 6.7 online service^97^ with the default background setting (i.e., with a background automatically selected by DAVID).

### Construction of prognostic gene signatures

The prognostic gene signatures were constructed using SVM^57^ with the linear kernel as described previously^40^. The triples with the highest prognostic power were identified by an exhaustive search with the threshold for sensitivity and specificity equal to 60% and 50% for the training and the filtration datasets, respectively. The triples that passed the filtration were further sorted in a descending order with respect to the mean value of AUC for the training and filtration datasets. The same procedure was used for the analysis of signatures with a different size (pairs, quadruples).

Construction of signatures for patients with ER-positive breast cancer utilized the collection of microarray datasets identical to the one we used previously^40^, as well as the previously used division into groups (recurrence within 5 years, no recurrence and follow-up of at least 7 years, and the remaining “gray zone”).

Construction of signatures for patients with colorectal cancer utilized the GSE39582 series^98^ as a training dataset, GSE37892^99^ and GSE17536^100,101^ as filtration datasets, and GSE14333^102^ as a validation (testing) dataset. Datasets were jointly preprocessed using RMA method^103^. Numbers of patients in these datasets are presented in Supplementary Table 6. At the training and filtration stages, only patients with recurrence within the first three years and patients without recurrence and follow-up of for at least four years were considered; other patients (comprising the so-called “grey zone”) were excluded from the analysis. Patients with an unknown recurrence status and patients with a recurrence within the first month were excluded from the analysis as well.

For the identified triples of genes with the highest prognostic power a 3-fold and 10-fold cross validation on the training dataset was performed as an additional test that proved an absence of overfitting. The results of cross-validation are presented in Supplementary Information (section *Cross-validation of the identified triples of genes on the training datasets*).

### Analysis of expression levels

Analysis of expression levels and expression variance of transcripts in the cell lines was performed using information from the RNA-Seq dataset E-MTAB-2706^56^, which contains genome-wide transcriptome profiles of 675 cancer cell lines. Reads per kilobase per million mapped reads (RPKM) metrics was used to represent the expression levels of genes; values of RPKM reported in the dataset file 140625_Klijn_RPKM_coding.txt were used. The variance in expression level for a given transcript was quantified as the third quartile to the first quartile ratio of expression levels for this transcript. With respect to the gene ranking, this quantification was equivalent to the quantification based on the interquartile range in log-scale. Transcripts that remained in the upper half after ranking by the first quartile of expression level had positive values of this quartile, and hence, the above quantification of expression variance was applicable for these transcripts.

Analysis of expression levels of genes in tissue specimens obtained from breast and colorectal cancer patients was performed similarly and utilized the following microarray datasets: GSE17705^104^ for breast cancer patients and GSE39582^98^ for colorectal cancer patients. These datasets were preprocessed using RMA method^103^ as parts of larger sets utilized for construction of prognostic gene signatures as described in the previous subsection. The analysis used expression levels reported by the RMA preprocessing algorithm.

### Statistical analysis

The significance level was set to 0.05. For Monte Carlo hypothesis testing, a conventional estimate of the p-value was used^105^. Log-rank tests were utilized to compare survival distribution between groups of patients; two-tailed p-values are reported. Binomial tests were used to analyze the size of intersection between halves or quarters of genes (with respect to a certain ranking) with the lists of genes comprising the network core.

### Data availability

The authors declare that the data supporting the findings of this study are available within the article and Supplementary Information or available from the authors upon request. All utilized datasets are properly referenced in the article and can be downloaded from publicly available databases.

## Electronic supplementary material

Supplementary Information

Supplementary Data
