# Supplementary material for: Comprehensive network of miRNA-induced intergenic interactions and a biological role of its core in cancer
Source: Sci Rep. 2018 Feb 5;8:2418. doi: 10.1038/s41598-018-20215-5 (PMC5799291; doi:10.1038/s41598-018-20215-5)
Supplement: Supplementary file 1 — Supplementary Information [file 41598_2018_20215_MOESM1_ESM.pdf]

Comprehensive network of miRNA-induced intergenic interactions and a biological role of its core in cancer

Vladimir V. Galatenko, Alexey V. Galatenko, Timur R. Samatov, Andrey A. Turchinovich, Maxim Yu. Shkurnikov, Julia A. Makarova, Alexander G. Tonevitsky

Supplementary Information

Network core: a simple example

In order to illustrate the definition of the network core, let us consider the following simple network.

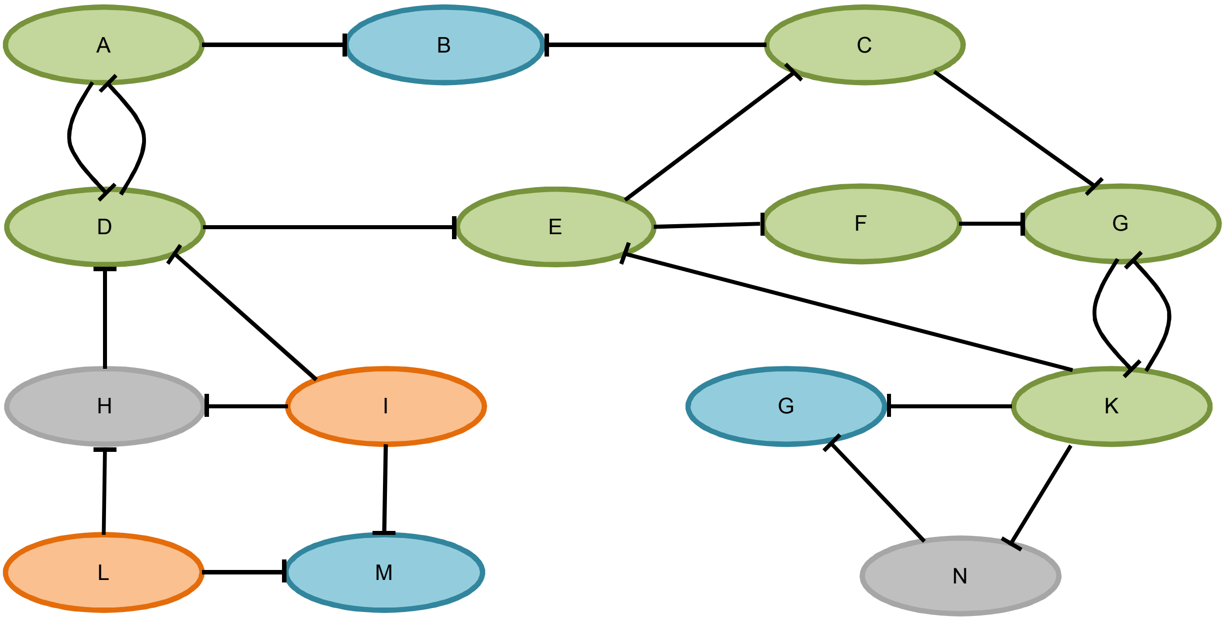

Supplementary Figure 1 | A simple example of a network. Nodes comprising the network core are colored in green. All these nodes have both in-edges and out-edges. The remaining nodes are colored in gray if they also have both in-edges and out-edges, in orange if they have only out-edges (and no in-edges), and in blue if they have only in-edges (and no out-edges). Overall the network contains 9 nodes with both in- and out-edges, 2 nodes with only out-edges and 3 nodes with only in-edges.

This network has two nontrivial (i.e., containing at least two nodes) strongly connected components: {A, D} and {C, E, F, G, K}. In each component every node is reachable from every other node via a directed path. E.g., node C is reachable from node F via a path

$$F \dashv G \dashv K \dashv E \dashv C.$$

Nodes B, G and N are reachable from the component {C, E, F, G, K}, but they do not belong to this component as the opposite reachability is violated (i.e., there is no directed path from these nodes to the nodes of the component). Similarly, the component {A, D} is reachable from nodes H, I and L, but these nodes can’t be added to the component due to absence of reverse reachability.

The enrichment analysis of lists of nodes with the highest in-degree

Lists of network nodes with in-degrees of at least 10 were formed. For the F-network, this list consisted of 142 genes (these are genes that are targeted by intragenic miRNAs of at least 10 other genes). For the IS-network, this list consisted of 90 genes (these are genes that are targeted by intronic sense miRNAs of at least 10 other genes).

Functional categories overrepresented in these lists were identified using DAVID 6.7 online service. These categories are presented in Supplementary Tables 1-4.

Supplementary Table 1 | Categories (terms) overrepresented in the list of genes with the highest in-degree in the F-network. All categories with Benjamini-corrected p-values less than 0.05 are listed.

Category	Term	Count	%	Fold Enrichment	P-Value	Benjamini
SP_PIR_KEYWORDS	Phosphoprotein	97	71.3	1.9	3.24×10^{-15}	6.63×10^{-13}
SP_PIR_KEYWORDS	Acetylation	47	34.6	2.5	1.40×10^{-9}	9.61×10^{-8}
SP_PIR_KEYWORDS	rna-binding	21	15.4	5.5	1.23×10^{-9}	1.27×10^{-7}
KEGG_PATHWAY	hsa04110:Cell cycle	11	8.1	9.3	1.35×10^{-7}	1.09×10^{-5}
KEGG_PATHWAY	hsa05220:Chronic myeloid leukemia	9	6.6	12.7	3.04×10^{-7}	1.23×10^{-5}
SP_PIR_KEYWORDS	Cytoplasm	49	36.0	2.1	2.68×10^{-7}	1.38×10^{-5}
KEGG_PATHWAY	hsa05200:Pathways in cancer	15	11.0	4.8	7.98×10^{-7}	2.15×10^{-5}
KEGG_PATHWAY	hsa05214:Glioma	8	5.9	13.5	1.36×10^{-6}	2.75×10^{-5}
KEGG_PATHWAY	hsa05219:Bladder cancer	7	5.1	17.7	1.83×10^{-6}	2.97×10^{-5}
KEGG_PATHWAY	hsa04115:p53 signaling pathway	8	5.9	12.5	2.30×10^{-6}	3.11×10^{-5}
SP_PIR_KEYWORDS	ubl conjugation	18	13.2	4.3	8.04×10^{-7}	3.31×10^{-5}
GOTERM_BP_FAT	GO:0010608~posttranscriptional regulation of gene expression	14	10.3	7.8	2.42×10^{-8}	3.32×10^{-5}
KEGG_PATHWAY	hsa05218:Melanoma	8	5.9	11.9	3.09×10^{-6}	3.58×10^{-5}
SP_PIR_KEYWORDS	nucleus	56	41.2	1.8	1.06×10^{-6}	3.62×10^{-5}
SP_PIR_KEYWORDS	cell cycle control	6	4.4	2<br>7.4	2.32×10^{-6}	6.83×10^{-5}
GOTERM_BP_FAT	GO:0010941~regulation of cell death	24	17.6	3.5	2.46×10^{-7}	8.42×10^{-5}
GOTERM_CC_FAT	GO:0000307~cyclin-dependent protein kinase holoenzyme complex	5	3.7	61.8	8.33×10^{-7}	9.83×10^{-5}
GOTERM_BP_FAT	GO:0043067~regulation of programmed cell death	24	17.6	3.5	2.31×10^{-7}	1.05×10^{-4}
SP_PIR_KEYWORDS	atp-binding	26	19.1	2.8	5.06×10^{-6}	1.30×10^{-4}
GOTERM_BP_FAT	GO:0042981~regulation of apoptosis	24	17.6	3.5	1.93×10^{-7}	1.32×10^{-4}
KEGG_PATHWAY	hsa05215:Prostate cancer	8	5.9	9.5	1.41×10^{-5}	1.43×10^{-4}
GOTERM_CC_FAT	GO:0031981~nuclear lumen	29	21.3	2.7	7.77×10^{-7}	1.83×10^{-4}
GOTERM_BP_FAT	GO:0010629~negative regulation of gene expression	18	13.2	4.2	1.03×10^{-6}	2.81×10^{-4}
GOTERM_CC_FAT	GO:0031974~membran×10-enclosed lumen	32	23.5	2.3	3.76×10^{-6}	2.95×10^{-4}
GOTERM_MF_FAT	GO:0003723~RNA binding	22	16.2	3.4	1.13×10^{-6}	3.42×10^{-4}
GOTERM_CC_FAT	GO:0043233~organelle lumen	31	22.8	2.3	7.57×10^{-6}	4.47×10^{-4}
GOTERM_BP_FAT	GO:0042127~regulation of cell proliferation	22	16.2	3.3	2.20×10^{-6}	5.02×10^{-4}
SP_PIR_KEYWORDS	cell cycle	14	10.3	4.3	2.24×10^{-5}	5.12×10^{-4}
SP_PIR_KEYWORDS	cyclin	6	4.4	17.0	2.60×10^{-5}	5.35×10^{-4}
KEGG_PATHWAY	hsa04510:Focal adhesion	10	7.4	5.3	7.27×10^{-5}	6.54×10^{-4}
SP_PIR_KEYWORDS	nucleotid×10-binding	28	20.6	2.3	3.85×10^{-5}	6.62×10^{-4}
GOTERM_CC_FAT	GO:0070013~intracellular organelle lumen	30	22.1	2.3	1.43×10^{-5}	6.73×10^{-4}
SP_PIR_KEYWORDS	Proto-oncogene	10	7.4	6.1	3.62×10^{-5}	6.78×10^{-4}
KEGG_PATHWAY	hsa05222:Small cell lung cancer	7	5.1	8.8	1.06×10^{-4}	8.59×10^{-4}
SP_PIR_KEYWORDS	repressor	13	9.6	4.2	5.79×10^{-5}	9.17×10^{-4}
GOTERM_BP_FAT	GO:0007167~enzyme linked receptor protein signaling pathway	14	10.3	4.8	5.97×10^{-6}	0.001165
SP_PIR_KEYWORDS	Apoptosis	12	8.8	4.5	7.96×10^{-5}	0.00117
GOTERM_BP_FAT	GO:0008284~positive regulation of cell proliferation	15	11.0	4.3	9.77×10^{-6}	0.00167
GOTERM_BP_FAT	GO:0010942~positive regulation of cell death	15	11.0	4.1	1.70×10^{-5}	0.001792
GOTERM_BP_FAT	GO:0009891~positive regulation of biosynthetic process	19	14.0	3.2	1.93×10^{-5}	0.001886
INTERPRO	IPR012677:Nucleotid×10-binding, alpha-beta plait	11	8.1	6.6	6.63×10^{-6}	0.001914
GOTERM_BP_FAT	GO:0048008~platelet-derived growth factor receptor signaling pathway	5	3.7	31.0	1.68×10^{-5}	0.001915
GOTERM_BP_FAT	GO:0043068~positive regulation of programmed cell death	15	11.0	4.1	1.62×10^{-5}	0.002011
GOTERM_BP_FAT	GO:0031328~positive regulation of cellular biosynthetic process	19	14.0	3.3	1.59×10^{-5}	0.002174
GOTERM_CC_FAT	GO:0005654~nucleoplasm	19	14.0	2.9	5.56×10^{-5}	0.002185
GOTERM_BP_FAT	GO:0043065~positive regulation of apoptosis	15	11.0	4.1	1.50×10^{-5}	0.002274
UP_SEQ_FEATURE	mutagenesis site	34	25.0	2.3	4.10×10^{-6}	0.002337

Category	Term	Count	%	Fold Enrichment	P-Value	Benjamini
GOTERM_BP_FAT	GO:0010605~negative regulation of macromolecule metabolic process	19	14.0	3.0	3.98×10^{-5}	0.003624
SP_PIR_KEYWORDS	ATP	9	6.6	5.4	2.65×10^{-4}	0.003636
GOTERM_BP_FAT	GO:0043066~negative regulation of apoptosis	13	9.6	4.3	4.30×10^{-5}	0.003671
GOTERM_BP_FAT	GO:0060548~negative regulation of cell death	13	9.6	4.2	5.06×10^{-5}	0.003839
SP_PIR_KEYWORDS	serine/threonin×10-protein kinase	11	8.1	4.1	3.62×10^{-4}	0.003923
SP_PIR_KEYWORDS	activator	13	9.6	3.5	3.05×10^{-4}	0.003924
UP_SEQ_FEATURE	nucleotide phosphat×10-binding region:ATP	21	15.4	3.1	1.38×10^{-5}	0.003938
GOTERM_BP_FAT	GO:0043069~negative regulation of programmed cell death	13	9.6	4.3	4.93×10^{-5}	0.003957
SP_PIR_KEYWORDS	Kinase	15	11.0	3.1	3.46×10^{-4}	0.003958
SP_PIR_KEYWORDS	transcription regulation	29	21.3	2.0	3.40×10^{-4}	0.004116
BIOCARTA	h_cellcyclePathway:Cyclins and Cell Cycle Regulation	6	4.4	11.5	9.39×10^{-5}	0.004356
SP_PIR_KEYWORDS	Transcription	29	21.3	2.0	4.86×10^{-4}	0.004991
GOTERM_BP_FAT	GO:0012502~induction of programmed cell death	12	8.8	4.4	8.27×10^{-5}	0.005129
GOTERM_MF_FAT	GO:0000166~nucleotide binding	38	27.9	1.9	6.97×10^{-5}	0.005266
GOTERM_BP_FAT	GO:0007049~cell cycle	19	14.0	2.9	8.18×10^{-5}	0.005312
GOTERM_MF_FAT	GO:0019904~protein domain specific binding	13	9.6	4.4	3.62×10^{-5}	0.005476
GOTERM_BP_FAT	GO:0006917~induction of apoptosis	12	8.8	4.4	8.04×10^{-5}	0.005486
GOTERM_BP_FAT	GO:0010604~positive regulation of macromolecule metabolic process	20	14.7	2.7	9.25×10^{-5}	0.005489
GOTERM_BP_FAT	GO:0022402~cell cycle process	16	11.8	3.3	7.76×10^{-5}	0.005571
GOTERM_BP_FAT	GO:0045449~regulation of transcription	40	29.4	1.8	1.09×10^{-4}	0.005699
GOTERM_MF_FAT	GO:0019899~enzyme binding	16	11.8	3.4	5.69×10^{-5}	0.005734
GOTERM_BP_FAT	GO:0016265~death	18	13.2	2.9	1.14×10^{-4}	0.005744
GOTERM_BP_FAT	GO:0010033~response to organic substance	18	13.2	2.9	1.08×10^{-4}	0.005899
GOTERM_BP_FAT	GO:0008219~cell death	18	13.2	2.9	1.05×10^{-4}	0.00594
INTERPRO	IPR000504:RNA recognition motif, RNP-1	10	7.4	6.0	4.15×10^{-5}	0.005973
BIOCARTA	h_RacCycDPathway:Influence of Ras and Rho proteins on G1 to S Transition	6	4.4	12.0	7.62×10^{-5}	0.007059
GOTERM_BP_FAT	GO:0051252~regulation of RNA metabolic process	31	22.8	2.0	1.68×10^{-4}	0.008167
GOTERM_BP_FAT	GO:0022403~cell cycle phase	13	9.6	3.7	1.90×10^{-4}	0.008617
GOTER_BP_FAT	GO:0012501~programmed cell death	16	11.8	3.1	1.84×10^{-4}	0.008655
GOTERM_BP_FAT	GO:0051329~interphase of mitotic cell cycle	7	5.1	8.0	2.31×10^{-4}	0.010141
GOTERM_BP_FAT	GO:0051325~interphase	7	5.1	7.8	2.70×10^{-4}	0.011477
SP_PIR_KEYWORDS	dna-binding	26	19.1	2.0	0.001186	0.011569
SP_PIR_KEYWORDS	translation regulation	5	3.7	10.6	0.001263	0.011767
GOTERM_BP_FAT	GO:0048534~hemopoietic or lymphoid organ development	10	7.4	4.5	3.49×10^{-4}	0.014346
GOTERM_BP_FAT	GO:0010557~positive regulation of macromolecule biosynthetic process	16	11.8	2.9	3.82×10^{-4}	0.015256
SP_PIR_KEYWORDS	isopeptide bond	9	6.6	4.0	0.001881	0.016719
GOTERM_BP_FAT	GO:0006468~protein amino acid phosphorylation	16	11.8	2.8	4.70×10^{-4}	0.018201
GOTERM_BP_FAT	GO:0016481~negative regulation of transcription	13	9.6	3.3	4.85×10^{-4}	0.018249
GOTERM_BP_FAT	GO:0002520~immune system development	10	7.4	4.3	5.38×10^{-4}	0.019183
GOTERM_BP_FAT	GO:0006915~apoptosis	15	11.0	2.9	5.28×10^{-4}	0.019335
GOTERM_BP_FAT	GO:0007169~transmembrane receptor protein tyrosine kinase signaling pathway	9	6.6	4.7	6.09×10^{-4}	0.021154
GOTERM_BP_FAT	GO:0032268~regulation of cellular protein metabolic process	13	9.6	3.2	6.44×10^{-4}	0.021802
SP_PIR_KEYWORDS	cell division	8	5.9	4.3	0.002644	0.022466
GOTERM_BP_FAT	GO:0007423~sensory organ development	9	6.6	4.6	7.04×10^{-4}	0.022684
GOTERM_BP_FAT	GO:0010558~negative regulation of macromolecule biosynthetic process	14	10.3	3.0	6.90×10^{-4}	0.022757
GOTERM_BP_FAT	GO:0045892~negative regulation of transcription, DNA-dependent	11	8.1	3.6	8.44×10^{-4}	0.026513
SMART	SM00360:RRM	10	7.4	4.5	3.21×10^{-4}	0.026592
GOTERM_BP_FAT	GO:0031327~negative regulation of cellular biosynthetic process	14	10.3	2.9	8.71×10^{-4}	0.026732
GOTERM_BP_FAT	GO:0051253~negative regulation of RNA metabolic process	11	8.1	3.6	9.59×10^{-4}	0.028741
GOTERM_BP_FAT	GO:0009890~negative regulation of biosynthetic process	14	10.3	2.9	0.001057	0.029099
SP_PIR_KEYWORDS	chromosomal rearrangement	8	5.9	4.1	0.003587	0.029173
GOTERM_BP_FAT	GO:0051173~positive regulation of nitrogen compound metabolic process	15	11.0	2.7	0.00102	0.029259
GOTERM_BP_FAT	GO:0000079~regulation of cyclin-dependent protein kinase activity	5	3.7	10.9	0.001093	0.029491
GOTERM_BP_FAT	GO:0006417~regulation of translation	7	5.1	6.0	0.001054	0.029596
GOTERM_BP_FAT	GO:0001889~liver development	5	3.7	11.1	0.001019	0.029866

Category	Term	Count	%	Fold Enrichment	P-Value	Benjamini
GOTERM_BP_FAT	GO:0000278~mitotic cell cycle	11	8.1	3.5	0.001131	0.029903
KEGG_PATHWAY	hsa05212:Pancreatic cancer	5	3.7	7.4	0.004164	0.030257
GOTERM_BP_FAT	GO:0045934~negative regulation of nucleobase, nucleoside, nucleotide and nucleic acid metabolic process	13	9.6	3.0	0.001257	0.031369
SP_PIR_KEYWORDS	host-virus interaction	8	5.9	4.0	0.004028	0.031475
GOTERM_BP_FAT	GO:0051270~regulation of cell motion	8	5.9	4.9	0.001228	0.031801
GOTERM_BP_FAT	GO:0016458~gene silencing	5	3.7	10.5	0.001254	0.031861
GOTERM_BP_FAT	GO:0000082~G1/S transition of mitotic cell cycle	5	3.7	10.5	0.001254	0.031861
GOTERM_BP_FAT	GO:0006355~regulation of transcription, DNA-dependent	28	20.6	1.9	0.001412	0.033927
GOTERM_BP_FAT	GO:0051172~negative regulation of nitrogen compound metabolic process	13	9.6	2.9	0.001411	0.034516
INTERPRO	IPR017442:Serine/threonine protein kinas×10-related	11	8.1	3.9	5.14×10^{-4}	0.036485
SP_PIR_KEYWORDS	protein kinase inhibitor	3	2.2	26.5	0.005501	0.041215
GOTERM_BP_FAT	GO:0006916~anti-apoptosis	8	5.9	4.6	0.001785	0.041964
INTERPRO	IPR008271:Serine/threonine protein kinase, active site	11	8.1	4.0	4.61×10^{-4}	0.043451
GOTERM_MF_FAT	GO:0004674~protein serine/threonine kinase activity	12	8.8	3.1	0.001493	0.044273
GOTERM_BP_FAT	GO:0043627~response to estrogen stimulus	6	4.4	6.7	0.001924	0.044409
GOTERM_MF_FAT	GO:0019900~kinase binding	8	5.9	5.0	0.001056	0.044722
GOTERM_MF_FAT	GO:0032559~adenyl ribonucleotide binding	26	19.1	1.9	0.001223	0.045299
GOTERM_BP_FAT	GO:0045768~positive regulation of anti-apoptosis	4	2.9	15.7	0.002005	0.045464
GOTERM_MF_FAT	GO:0030528~transcription regulator activity	26	19.1	1.9	0.001409	0.046366
KEGG_PATHWAY	hsa05210:Colorectal cancer	5	3.7	6.3	0.007204	0.04763
GOTERM_BP_FAT	GO:0060541~respiratory system development	6	4.4	6.5	0.002177	0.048482
INTERPRO	IPR017441:Protein kinase, ATP binding site	12	8.8	3.4	8.79×10^{-4}	0.049551
KEGG_PATHWAY	hsa04012:ErbB signaling pathway	5	3.7	6.1	0.008145	0.04968
GOTERM_MF_FAT	GO:0005524~ATP binding	26	19.1	2.0	0.001009	0.049687

Supplementary Table 2 | Categories (terms) overrepresented in the list of genes with the highest in-degree in the IS-network. All categories with Benjamini-corrected p-values less than 0.05 are listed.

Category	Term	Count	%	Fold Enrichment	P-Value	Benjamini
SP_PIR_KEYWORDS	phosphoprotein	60	69.8	1.8	3.07×10^{-9}	4.79×10^{-7}
KEGG_PATHWAY	hsa04110:Cell cycle	9	10.5	12.6	2.19×10^{-7}	1.16×10^{-5}
KEGG_PATHWAY	hsa04115:p53 signaling pathway	7	8.1	18.1	1.37×10^{-6}	3.62×10^{-5}
SP_PIR_KEYWORDS	Cytoplasm	34	39.5	2.3	2.09×10^{-6}	1.09×10^{-4}
SP_PIR_KEYWORDS	ubl conjugation	14	16.3	5.3	1.75×10^{-6}	1.37×10^{-4}
KEGG_PATHWAY	hsa05214:Glioma	6	7.0	16.7	1.96×10^{-5}	3.46×10^{-4}
KEGG_PATHWAY	hsa05220:Chronic myeloid leukemia	6	7.0	14.0	4.60×10^{-5}	4.06×10^{-4}
KEGG_PATHWAY	hsa05200:Pathways in cancer	10	11.6	5.3	4.00×10^{-5}	4.24×10^{-4}
KEGG_PATHWAY	hsa05218:Melanoma	6	7.0	14.8	3.52×10^{-5}	4.67×10^{-4}
KEGG_PATHWAY	hsa05222:Small cell lung cancer	6	7.0	12.5	7.95×10^{-5}	4.68×10^{-4}
KEGG_PATHWAY	hsa04510:Focal adhesion	8	9.3	7.0	7.92×10^{-5}	5.25×10^{-4}
KEGG_PATHWAY	hsa05219:Bladder cancer	5	5.8	20.9	7.13×10^{-5}	5.40×10^{-4}
KEGG_PATHWAY	hsa05215:Prostate cancer	6	7.0	11.8	1.05×10^{-4}	5.56×10^{-4}
UP_SEQ_FEATURE	mutagenesis site	26	30.2	2.8	1.71×10^{-6}	7.02×10^{-4}
SP_PIR_KEYWORDS	nucleus	37	43.0	1.9	2.70×10^{-5}	8.44×10^{-4}
SP_PIR_KEYWORDS	rna-binding	12	14.0	5.0	2.52×10^{-5}	9.81×10^{-4}
GOTERM_BP_FAT	GO:0010941~regulation of cell death	17	19.8	3.9	4.43×10^{-6}	0.001078
SP_PIR_KEYWORDS	Apoptosis	10	11.6	5.9	4.67×10^{-5}	0.001212
GOTERM_BP_FAT	GO:0043067~regulation of programmed cell death	17	19.8	3.9	4.23×10^{-6}	0.001371
SP_PIR_KEYWORDS	cyclin	5	5.8	22.4	7.00×10^{-5}	0.00156
GOTERM_BP_FAT	GO:0010608~posttranscriptional regulation of gene expression	10	11.6	8.8	1.67×10^{-6}	0.00163
GOTERM_BP_FAT	GO:0042981~regulation of apoptosis	17	19.8	3.9	3.72×10^{-6}	0.00181
GOTERM_CC_FAT	GO:0000307~cyclin-dependent protein kinase holoenzyme complex	4	4.7	80.1	1.35×10^{-5}	0.002054
GOTERM_BP_FAT	GO:0042127~regulation of cell proliferation	16	18.6	3.8	1.33×10^{-5}	0.002585
SP_PIR_KEYWORDS	atp-binding	17	19.8	2.9	2.01×10^{-4}	0.003483
GOTERM_BP_FAT	GO:0010033~response to organic substance	15	17.4	3.9	2.20×10^{-5}	0.003569
SP_PIR_KEYWORDS	cell cycle	10	11.6	4.9	1.98×10^{-4}	0.003857
SP_PIR_KEYWORDS	cell cycle control	4	4.7	28.9	3.42×10^{-4}	0.005327
GOTERM_BP_FAT	GO:0060548~negative regulation of cell death	10	11.6	5.1	1.17×10^{-4}	0.007128
GOTERM_BP_FAT	GO:0010942~positive regulation of cell death	11	12.8	4.7	9.60×10^{-5}	0.007165

Category	Term	Count	%	Fold Enrichment	P-Value	Benjamini
GOTERM_BP_FAT	GO:0043066~negative regulation of apoptosis	10	11.6	5.2	$1.03{\times}10^{-4}$	0.00717
GOTERM_BP_FAT	GO:0043069~negative regulation of programmed cell death	10	11.6	5.2	$1.15{\times}10^{-4}$	0.007443
GOTERM_BP_FAT	GO:0043068~positive regulation of programmed cell death	11	12.8	4.7	$9.24{\times}10^{-5}$	0.00747
GOTERM_BP_FAT	GO:0048008~platelet-derived growth factor receptor signaling pathway	4	4.7	39.0	$1.32{\times}10^{-4}$	0.007522
GOTERM_BP_FAT	GO:0043065~positive regulation of apoptosis	11	12.8	4.7	$8.72{\times}10^{-5}$	0.007693
SP_PIR_KEYWORDS	Proto-oncogene	7	8.1	6.8	$5.45{\times}10^{-4}$	0.007704
GOTERM_BP_FAT	GO:0012501~programmed cell death	13	15.1	3.9	$8.27{\times}10^{-5}$	0.008027
GOTERM_BP_FAT	GO:0010629~negative regulation of gene expression	12	14.0	4.4	$6.64{\times}10^{-5}$	0.008052
SP_PIR_KEYWORDS	ATP	7	8.1	6.6	$6.24{\times}10^{-4}$	0.008083
GOTERM_BP_FAT	GO:0007167~enzyme linked receptor protein signaling pathway	10	11.6	5.4	$7.95{\times}10^{-5}$	0.008565
GOTERM_BP_FAT	GO:0008284~positive regulation of cell proliferation	11	12.8	4.9	$6.36{\times}10^{-5}$	0.008811
SP_PIR_KEYWORDS	kinase	11	12.8	3.6	$8.95{\times}10^{-4}$	0.009933
SP_PIR_KEYWORDS	acetylation	24	27.9	2.0	$8.38{\times}10^{-4}$	0.010014
GOTERM_BP_FAT	GO:0000079~regulation of cyclin-dependent protein kinase activity	5	5.8	17.2	$1.91{\times}10^{-4}$	0.010271
GOTERM_BP_FAT	GO:0051329~interphase of mitotic cell cycle	6	7.0	10.8	$2.17{\times}10^{-4}$	0.011048
SP_PIR_KEYWORDS	dna-binding	19	22.1	2.3	0.001165	0.011305
SP_PIR_KEYWORDS	transcription regulation	20	23.3	2.2	0.001147	0.011867
GOTERM_BP_FAT	GO:0051325~interphase	6	7.0	10.5	$2.48{\times}10^{-4}$	0.011995
SP_PIR_KEYWORDS	Transcription	20	23.3	2.2	0.001489	0.012827
SP_PIR_KEYWORDS	serine/threonin×10-protein kinase	8	9.3	4.7	0.001485	0.013547
GOTERM_BP_FAT	GO:0006915~apoptosis	12	14.0	3.7	$3.16{\times}10^{-4}$	0.014535
GOTERM_BP_FAT	GO:0051252~regulation of RNA metabolic process	22	25.6	2.2	$3.46{\times}10^{-4}$	0.015225
GOTERM_BP_FAT	GO:0008219~cell death	13	15.1	3.4	$3.76{\times}10^{-4}$	0.015817
GOTERM_BP_FAT	GO:0016265~death	13	15.1	3.3	$4.01{\times}10^{-4}$	0.016137
BIOCARTA	h_cellcyclePathway:Cyclins and Cell Cycle Regulation	5	5.8	13.7	$2.87{\times}10^{-4}$	0.016217
SP_PIR_KEYWORDS	activator	9	10.5	3.9	0.002102	0.017129
SP_PIR_KEYWORDS	protein kinase inhibitor	3	3.5	41.9	0.002225	0.017221
GOTERM_BP_FAT	GO:0010605~negative regulation of macromolecule metabolic process	13	15.1	3.3	$4.54{\times}10^{-4}$	0.017519
SP_PIR_KEYWORDS	nucleotid×10-binding	17	19.8	2.3	0.002665	0.019626
GOTERM_BP_FAT	GO:0050678~regulation of epithelial cell proliferation	5	5.8	13.1	$5.48{\times}10^{-4}$	0.02031
SP_PIR_KEYWORDS	isopeptide bond	7	8.1	4.9	0.002897	0.02036
SP_PIR_KEYWORDS	translation regulation	4	4.7	13.4	0.003254	0.021864
GOTERM_BP_FAT	GO:0022402~cell cycle process	11	12.8	3.6	$7.75{\times}10^{-4}$	0.02486
GOTERM_BP_FAT	GO:0000278~mitotic cell cycle	9	10.5	4.5	$7.55{\times}10^{-4}$	0.025039
GOTERM_BP_FAT	GO:0009725~response to hormone stimulus	9	10.5	4.5	$7.16{\times}10^{-4}$	0.025497
GOTERM_BP_FAT	GO:0007049~cell cycle	13	15.1	3.1	$7.44{\times}10^{-4}$	0.025569
GOTERM_CC_FAT	GO:0031981~nuclear lumen	17	19.8	2.6	$4.31{\times}10^{-4}$	0.032249
GOTERM_BP_FAT	GO:0007169~transmembrane receptor protein tyrosine kinase signaling pathway	7	8.1	5.8	0.001212	0.037377
SP_PIR_KEYWORDS	rna-mediated gene silencing	3	3.5	24.9	0.006305	0.038699
GOTERM_BP_FAT	GO:0045449~regulation of transcription	26	30.2	1.9	0.001389	0.040185
SP_PIR_KEYWORDS	cell division	6	7.0	5.1	0.006305	0.040278
GOTERM_BP_FAT	GO:0009719~response to endogenous stimulus	9	10.5	4.1	0.001351	0.0403
GOTERM_CC_FAT	GO:0031974~membran×10-enclosed lumen	19	22.1	2.3	$8.24{\times}10^{-4}$	0.040899
GOTERM_BP_FAT	GO:0012502~induction of programmed cell death	8	9.3	4.6	0.00156	0.041362
GOTERM_MF_FAT	GO:0019899~enzyme binding	11	12.8	4.0	$3.66{\times}10^{-4}$	0.041436
GOTERM_BP_FAT	GO:0022403~cell cycle phase	9	10.5	4.0	0.001552	0.042316
GOTERM_BP_FAT	GO:0006917~induction of apoptosis	8	9.3	4.6	0.001532	0.042984
GOTERM_BP_FAT	GO:0010604~positive regulation of macromolecule metabolic process	13	15.1	2.8	0.001754	0.045158
GOTERM_BP_FAT	GO:0030324~lung development	5	5.8	9.4	0.001897	0.045184
GOTERM_BP_FAT	GO:0051726~regulation of cell cycle	8	9.3	4.5	0.001858	0.045383
GOTERM_BP_FAT	GO:0006355~regulation of transcription, DNA-dependent	20	23.3	2.1	0.001853	0.046432
SP_PIR_KEYWORDS	chromosomal rearrangement	6	7.0	4.8	0.007913	0.04655
GOTERM_BP_FAT	GO:0030323~respiratory tube development	5	5.8	9.1	0.002116	0.049078

Supplementary Table 3 | Top five clusters of categories (terms) overrepresented in the list of genes with the highest in-degree in the F-network.

**Annotation Cluster 1**	**Enrichment Score: 4.76**					
**Category**	**Term**	**Count**	**%**	**Fold Enrichment**	**P-Value**	**Benjamini**
GOTERM_CC_FAT	GO:0031981~nuclear lumen	29	21.3	2.7	7.77×10^{-7}	1.83×10^{-4}
GOTERM_CC_FAT	GO:0031974~membrane-enclosed lumen	32	23.5	2.3	3.76×10^{-6}	2.95×10^{-4}
GOTERM_CC_FAT	GO:0043233~organelle lumen	31	22.8	2.3	7.57×10^{-6}	4.47×10^{-4}
GOTERM_CC_FAT	GO:0070013~intracellular organelle lumen	30	22.1	2.3	1.43×10^{-5}	6.73×10^{-4}
GOTERM_CC_FAT	GO:0005654~nucleoplasm	19	14.0	2.9	5.56×10^{-5}	0.002185
GOTERM_CC_FAT	GO:0005730~nucleolus	14	10.3	2.7	0.001567	0.051489

**Annotation Cluster 2**	**Enrichment Score: 4.55**					
**Category**	**Term**	**Count**	**%**	**Fold Enrichment**	**PValue**	**Benjamini**
GOTERM_BP_FAT	GO:0042981~regulation of apoptosis	24	17.6	3.5	1.93×10^{-7}	1.32×10^{-4}
GOTERM_BP_FAT	GO:0043067~regulation of programmed cell death	24	17.6	3.5	2.31×10^{-7}	1.05×10^{-4}
GOTERM_BP_FAT	GO:0010941~regulation of cell death	24	17.6	3.5	2.46×10^{-7}	8.42×10^{-5}
GOTERM_BP_FAT	GO:0043065~positive regulation of apoptosis	15	11.0	4.1	1.50×10^{-5}	0.002274
GOTERM_BP_FAT	GO:0043068~positive regulation of programmed cell death	15	11.0	4.1	1.62×10^{-5}	0.002011
GOTERM_BP_FAT	GO:0010942~positive regulation of cell death	15	11.0	4.1	1.70×10^{-5}	0.001792
GOTERM_BP_FAT	GO:0043066~negative regulation of apoptosis	13	9.6	4.3	4.30×10^{-5}	0.003671
GOTERM_BP_FAT	GO:0043069~negative regulation of programmed cell death	13	9.6	4.3	4.93×10^{-5}	0.003957
GOTERM_BP_FAT	GO:0060548~negative regulation of cell death	13	9.6	4.2	5.06×10^{-5}	0.003839
SP_PIR_KEYWORDS	Apoptosis	12	8.8	4.5	7.96×10^{-5}	0.00117
GOTERM_BP_FAT	GO:0006917~induction of apoptosis	12	8.8	4.4	8.04×10^{-5}	0.005486
GOTERM_BP_FAT	GO:0012502~induction of programmed cell death	12	8.8	4.4	8.27×10^{-5}	0.005129
GOTERM_BP_FAT	GO:0008219~cell death	18	13.2	2.9	1.05×10^{-4}	0.00594
GOTERM_BP_FAT	GO:0016265~death	18	13.2	2.9	1.14×10^{-4}	0.005744
GOTERM_BP_FAT	GO:0012501~programmed cell death	16	11.8	3.1	1.84×10^{-4}	0.008655
GOTERM_BP_FAT	GO:0006915~apoptosis	15	11.0	2.9	5.28×10^{-4}	0.019335
GOTERM_BP_FAT	GO:0006916~anti-apoptosis	8	5.9	4.6	0.001785	0.041964

**Annotation Cluster 3**	**Enrichment Score: 4.17**					
**Category**	**Term**	**Count**	**%**	**Fold Enrichment**	**PValue**	**Benjamini**
GOTERM_BP_FAT	GO:0010608~posttranscriptional regulation of gene expression	14	10.3	7.8	2.42×10^{-8}	3.32×10^{-5}
GOTERM_BP_FAT	GO:0032268~regulation of cellular protein metabolic process	13	9.6	3.2	6.44×10^{-4}	0.021802
GOTERM_BP_FAT	GO:0006417~regulation of translation	7	5.1	6.0	0.001054	0.029596
SP_PIR_KEYWORDS	translation regulation	5	3.7	10.6	0.001263	0.011767

**Annotation Cluster 4**	**Enrichment Score: 4.06**					
**Category**	**Term**	**Count**	**%**	**Fold Enrichment**	**PValue**	**Benjamini**
SP_PIR_KEYWORDS	rna-binding	21	15.4	5.5	1.23×10^{-9}	1.27×10^{-7}
GOTERM_MF_FAT	GO:0003723~RNA binding	22	16.2	3.4	1.13×10^{-6}	3.42×10^{-4}
INTERPRO	IPR012677:Nucleotide-binding, alpha-beta plait	11	8.1	6.6	6.63×10^{-6}	0.001914
INTERPRO	IPR000504:RNA recognition motif, RNP-1	10	7.4	6.0	4.15×10^{-5}	0.005973
SMART	SM00360:RRM	10	7.4	4.5	3.21×10^{-4}	0.026592
UP_SEQ_FEATURE	domain:RRM	6	4.4	7.1	0.001566	0.13857
UP_SEQ_FEATURE	domain:RRM 2	5	3.7	7.0	0.00573	0.279738
UP_SEQ_FEATURE	domain:RRM 1	5	3.7	7.0	0.00573	0.279738
UP_SEQ_FEATURE	domain:RRM 3	3	2.2	8.4	0.04867	0.794592

**Annotation Cluster 5**	**Enrichment Score: 3.61**					
**Category**	**Term**	**Count**	**%**	**Fold Enrichment**	**PValue**	**Benjamini**
GOTERM_BP_FAT	GO:0007167~enzyme linked receptor protein signaling pathway	14	10.3	4.8	5.97×10^{-6}	0.001165
GOTERM_BP_FAT	GO:0048008~platelet-derived growth factor receptor signaling pathway	5	3.7	31.0	1.68×10^{-5}	0.001915
GOTERM_BP_FAT	GO:0007169~transmembrane receptor protein tyrosine kinase signaling pathway	9	6.6	4.7	6.09×10^{-4}	0.021154
GOTERM_BP_FAT	GO:0007166~cell surface receptor linked signal transduction	23	16.9	1.5	0.059357	0.43637

Supplementary Table 4 | Top three clusters of categories (terms) overrepresented in the list of genes with the highest in-degree in the IS-network.

**Annotation Cluster 1**	**Enrichment Score: 3.89**					
**Category**	**Term**	**Count**	**%**	**Fold Enrichment**	**P-Value**	**Benjamini**
GOTERM_BP_FAT	GO:0042981~regulation of apoptosis	17	19.8	3.9	3.72×10^{-6}	0.00181
GOTERM_BP_FAT	GO:0043067~regulation of programmed cell death	17	19.8	3.9	4.23×10^{-6}	0.001371
GOTERM_BP_FAT	GO:0010941~regulation of cell death	17	19.8	3.9	4.43×10^{-6}	0.001078
SP_PIR_KEYWORDS	Apoptosis	10	11.6	5.9	4.67×10^{-5}	0.001212
GOTERM_BP_FAT	GO:0012501~programmed cell death	13	15.1	3.9	8.27×10^{-5}	0.008027
GOTERM_BP_FAT	GO:0043065~positive regulation of apoptosis	11	12.8	4.7	8.72×10^{-5}	0.007693
GOTERM_BP_FAT	GO:0043068~positive regulation of programmed cell death	11	12.8	4.7	9.24×10^{-5}	0.00747
GOTERM_BP_FAT	GO:0010942~positive regulation of cell death	11	12.8	4.7	9.60×10^{-5}	0.007165
GOTERM_BP_FAT	GO:0043066~negative regulation of apoptosis	10	11.6	5.2	1.03×10^{-4}	0.00717
GOTERM_BP_FAT	GO:0043069~negative regulation of programmed cell death	10	11.6	5.2	1.15×10^{-4}	0.007443
GOTERM_BP_FAT	GO:0060548~negative regulation of cell death	10	11.6	5.1	1.17×10^{-4}	0.007128
GOTERM_BP_FAT	GO:0006915~apoptosis	12	14.0	3.7	3.16×10^{-4}	0.014535
GOTERM_BP_FAT	GO:0008219~cell death	13	15.1	3.4	3.76×10^{-4}	0.015817
GOTERM_BP_FAT	GO:0016265~death	13	15.1	3.3	4.01×10^{-4}	0.016137
GOTERM_BP_FAT	GO:0006917~induction of apoptosis	8	9.3	4.6	0.001532	0.042984
GOTERM_BP_FAT	GO:0012502~induction of programmed cell death	8	9.3	4.6	0.00156	0.041362
GOTERM_BP_FAT	GO:0006916~anti-apoptosis	5	5.8	4.5	0.024044	0.238507

**Annotation Cluster 2**	**Enrichment Score: 3.04**					
**Category**	**Term**	**Count**	**%**	**Fold Enrichment**	**P-Value**	**Benjamini**
GOTERM_BP_FAT	GO:0010608~posttranscriptional regulation of gene expression	10	11.6	8.8	1.67×10^{-6}	0.00163
SP_PIR_KEYWORDS	translation regulation	4	4.7	13.4	0.003254	0.021864
GOTERM_BP_FAT	GO:0032268~regulation of cellular protein metabolic process	9	10.5	3.5	0.00359	0.06514
GOTERM_BP_FAT	GO:0006417~regulation of translation	4	4.7	5.4	0.036521	0.30398

**Annotation Cluster 3**	**Enrichment Score: 2.96**					
**Category**	**Term**	**Count**	**%**	**Fold Enrichment**	**P-Value**	**Benjamini**
GOTERM_BP_FAT	GO:0007167~enzyme linked receptor protein signaling pathway	10	11.6	5.4	7.95×10^{-5}	0.008565
GOTERM_BP_FAT	GO:0048008~platelet-derived growth factor receptor signaling pathway	4	4.7	39.0	1.32×10^{-4}	0.007522
GOTERM_BP_FAT	GO:0007169~transmembrane receptor protein tyrosine kinase signaling pathway	7	8.1	5.8	0.001212	0.037377
GOTERM_BP_FAT	GO:0007166~cell surface receptor linked signal transduction	15	17.4	1.5	0.109849	0.568095

Three-node sub-networks

In total there exists sixteen non-equivalent (i.e., non-isomorphic) directed three-node sub-networks. All of them are shown in Supplementary Fig. 2. Three of these sub-networks are disconnected, and thirteen are connected. For all connected sub-networks we summarized data on their occurrence in random graphs as well as in the constructed F-network and IS-network in Supplementary Table 5.

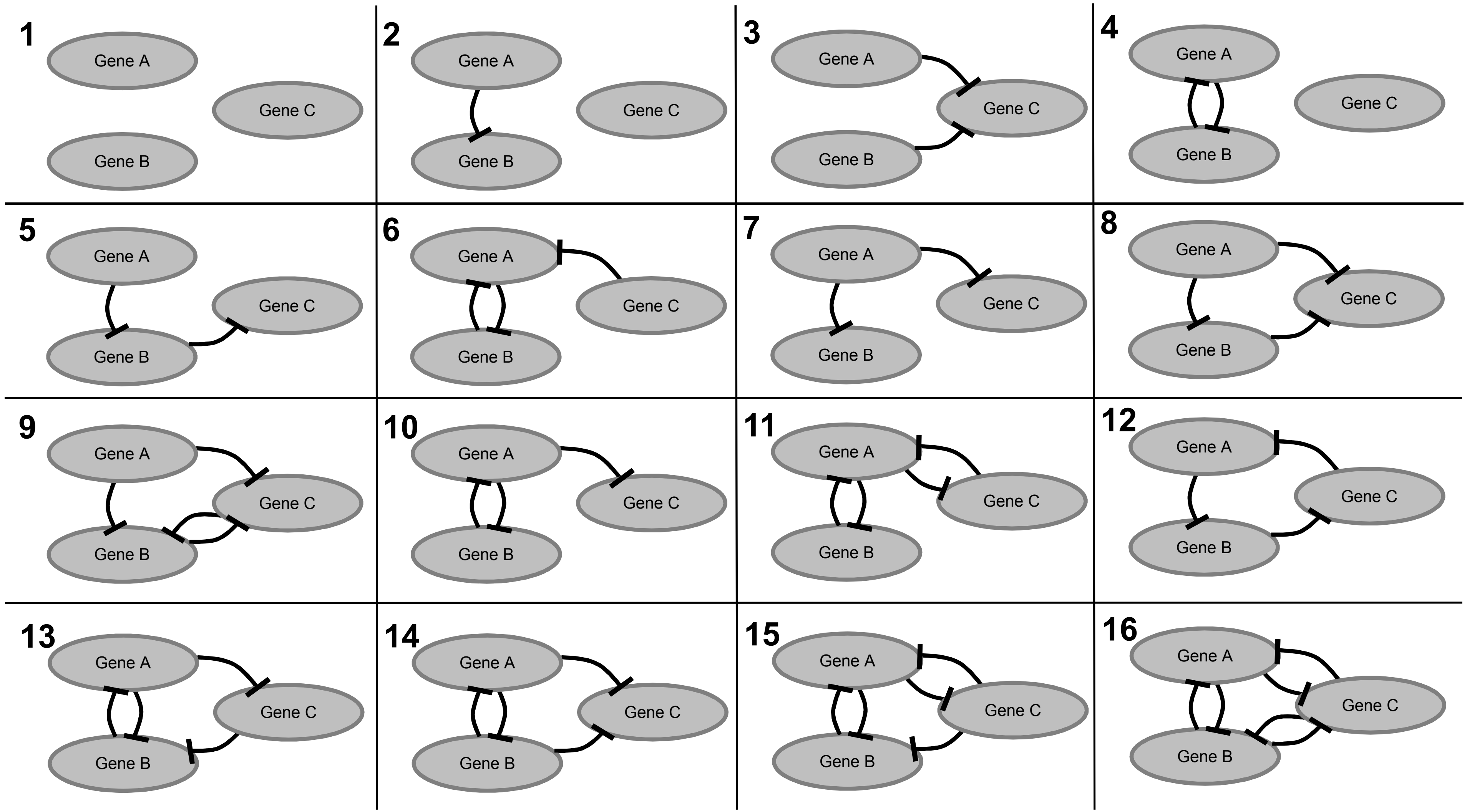

Supplementary Figure 2 | All non-equivalent (i.e., non-isomorphic) directed three-node sub-networks. Sub-networks 1, 2, 4 are disconnected, all other sub-networks are connected.

Supplementary Table 5 | Aggregate information on number of connected three-node sub-networks of each type in the F- and IS-networks and in generated random graphs. Sub-network types are given in accordance with Supplementary Fig. 2. For random graphs interquartile intervals [Q1, Q3] for numbers of sub-networks are specified. If an observed number of a sub-network does not belong to the interquartile interval, the conventional estimate of the p-value[1] is additionally presented below the interquartile interval.

Type	*IS-network*			*F-network*		
	Observed	*Erdős–Rényi*	*Fixed out-degree*	*Observed*	*Erdős–Rényi*	*Fixed out-degree*
3	**22913**	**[17109, 17282]<br>$<10^{-4}$**	**[12907, 13610]<br>$<10^{-4}$**	**30757**	**[21178, 21374]<br>$<10^{-4}$**	**[16479, 17208]<br>$<10^{-4}$**
5	24917	**[34268, 34521]<br>$<10^{-4}$**	[23734, 29111]	32654	**[42418, 42695]<br>$<10^{-4}$**	[30554, 36626]
6	**18**	[4, 12]<br>0.10	**[2, 8]<br>0.017**	**41**	**[5, 16]<br>0.0026**	**[3, 11]<br>$<10^{-4}$**
7	7293634	**[17110, 17286]<br>$<10^{-4}$**	[7289687, 7293494]<br>0.23	7732631	**[21182, 21377]<br>$<10^{-4}$**	[7732075, 7736051]
8	1948	**[6, 10]<br>$<10^{-4}$**	**[2587, 3269]<br>0.016**	2448	**[9, 13]<br>$<10^{-4}$**	**[3049, 3739]<br>0.025**
9	0	[0, 0]	[0, 0]	0	[0, 0]	[0, 1]
10	1773	**[4, 12]<br>$<10^{-4}$**	[928, 4389]	5333	**[5, 16]<br>$<10^{-4}$**	[1341, 4969]<br>0.22
11	0	[0, 0]	[0, 0]	2	**[0, 0]<br>$<10^{-4}$**	[0, 1]<br>0.067
12	0	[2, 4]<br>0.065	[1, 3]<br>0.21	0	**[2, 5]<br>0.025**	[1, 4]<br>0.12
13	0	[0, 0]	[0, 1]	2	**[0, 0]<br>$<10^{-4}$**	[0, 1]<br>0.18
14	**53**	**[0, 0]<br>$<10^{-4}$**	[7, 216]	**248**	**[0, 0]<br>$<10^{-4}$**	[16, 228]<br>0.22
15	0	[0, 0]	[0, 0]	0	[0, 0]	[0, 0]
16	0	[0, 0]	[0, 0]	0	[0, 0]	[0, 0]

Histograms supporting p-values

Histograms supporting specified p-values associated with the three-node network motif depicted in Fig. 2a (sub-network type 6 in accordance with Supplementary Fig. 2) are presented in Supplementary Fig. 3.

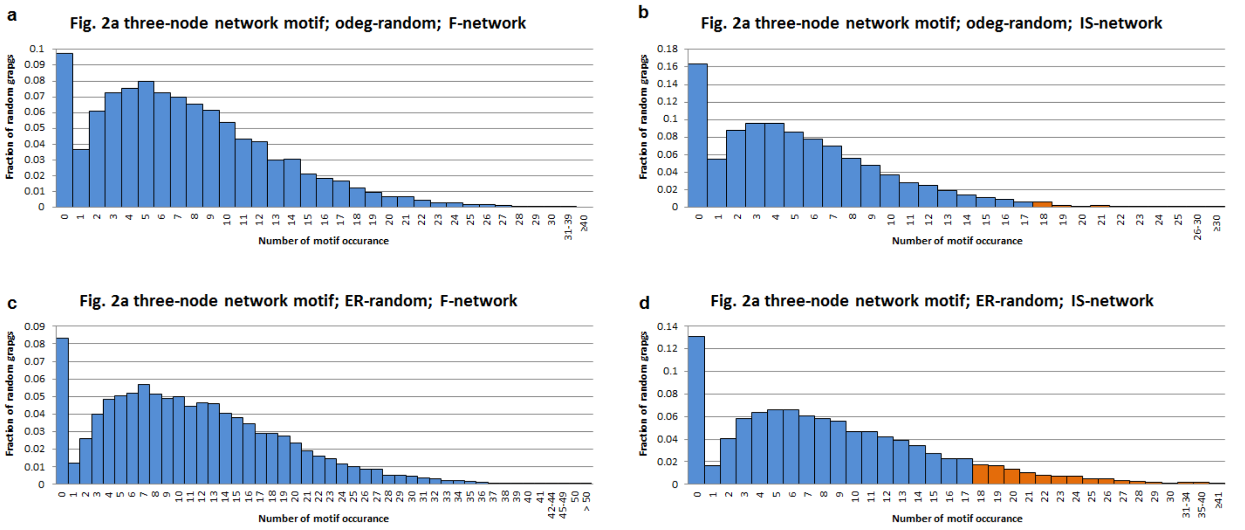

Supplementary Figure 3 | Histograms supporting specified p-values associated with the network motif shown in Fig. 2a (sub-network type 6 in accordance with Supplementary Fig. 2). Bars for which the value is equal to or greater than the number of occurrence observed in the constructed networks (i.e., bars contributing to p-value) are colored in orange. Panels **a**, **b** correspond to the model of random graph generation with the fixed out-degrees, and panels **c**, **d** correspond to the Erdős–Rényi model. Panels **a**, **c** correspond to the F-network, and panels **b**, **d** correspond to the IS-network.

For the network motif depicted in Fig. 2b (sub-network type 14 in accordance with Supplementary Fig. 2) in case of the F-network the Erdős–Rényi model generated 9,983 random graphs with no occurrence of this sub-network and 17 random graphs with one occurrence; no graphs with at least two occurrences were generated. The numbers for the IS-network were similar (9,988 and 12; no graphs with at least two occurrences).

For the network motif depicted in Fig. 2c (sub-network type 3 in accordance with Supplementary Fig. 2) the number of occurrence was 30,757 for the F-network and 22,913 for the IS-network (see Supplementary Table 5). These numbers exceed the number of occurrence in all generated random graphs. For the Erdős–Rényi model histograms for random graphs are presented in Supplementary Fig. 4. For the model with the fixed out-degrees the difference was even more striking (maximum observed numbers of occurrence in random graphs were 18,568 and 14,846 for the F-network and for the IS-network, respectively).

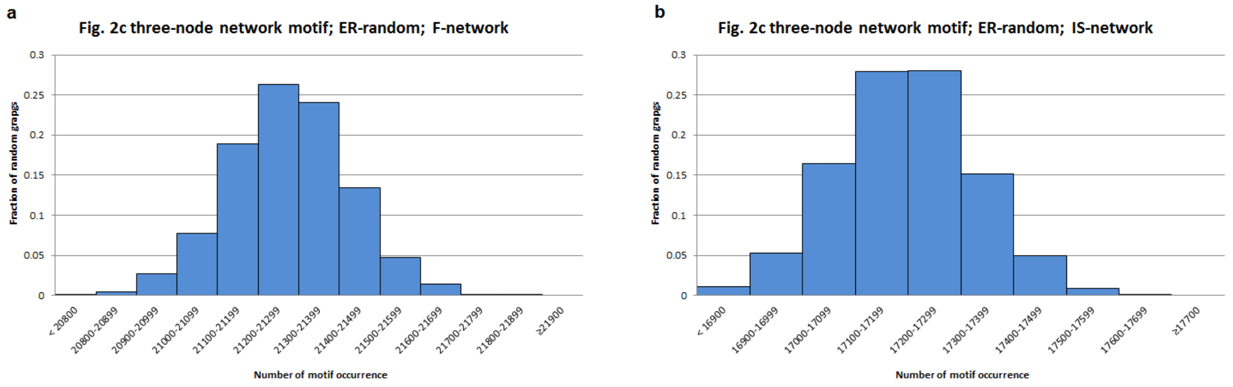

Supplementary Figure 4 | Histograms supporting specified p-values associated with the network motif shown in Fig. 2c (sub-network type 3 in accordance with Supplementary Fig. 2) for the Erdős–Rényi model. Panel **a** corresponds to the F-network, and panel **b** corresponds to the IS-network.

In case of the Erdős–Rényi model random graph core size exceeded 5,000 for all the generated graphs (both for the F-network and the IS-network). Histograms for the core size of random graphs generated by the model with the fixed out-degrees are presented in Supplementary Fig. 5.

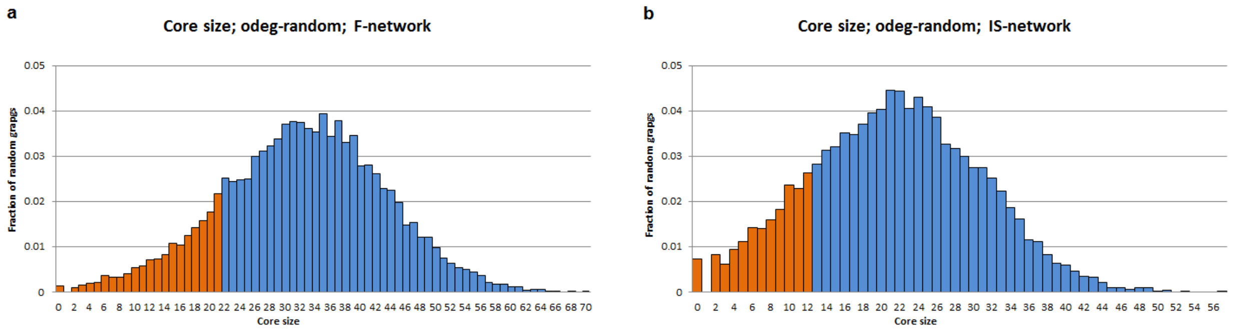

Supplementary Figure 5 | Histograms supporting specified p-values associated with the network core size (the model with the fixed out-degrees). Panel **a** corresponds to the F-network, and panel b corresponds to the IS-network. Bars for which the value is equal to or less than the core size in the constructed networks (i.e., bars contributing to p-value) are colored in orange.

The core of the F-network

Genes forming the core of the F-network and intergenic interaction between these genes induced by the intragenic miRNAs are presented in Supplementary Fig. 6.

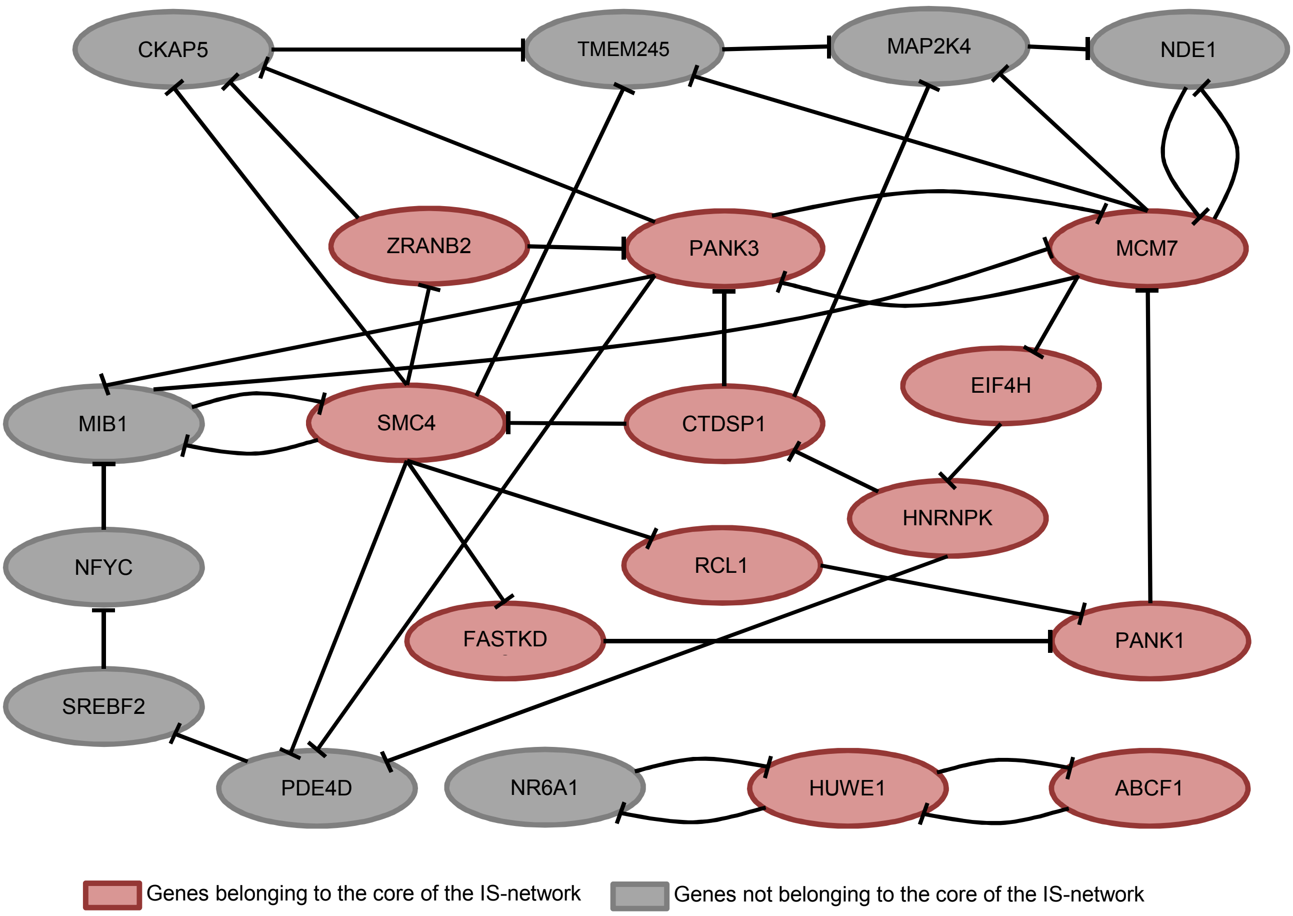

Supplementary Figure 6 | The core of the F-network. The nodes are genes, and the nodes (genes) *A* and *B* are connected by a directed edge (*A*—| *B*) if gene *A* hosts a miRNA that targets gene *B*.

Boxplots for the expression levels of genes in the collection of cell lines

For the analyzed collection of 675 cell lines, boxplots illustrating the data on higher expression levels and lower expression variance of genes comprising the network core (compared to the median values) are presented in Supplementary Fig. 7, 8.

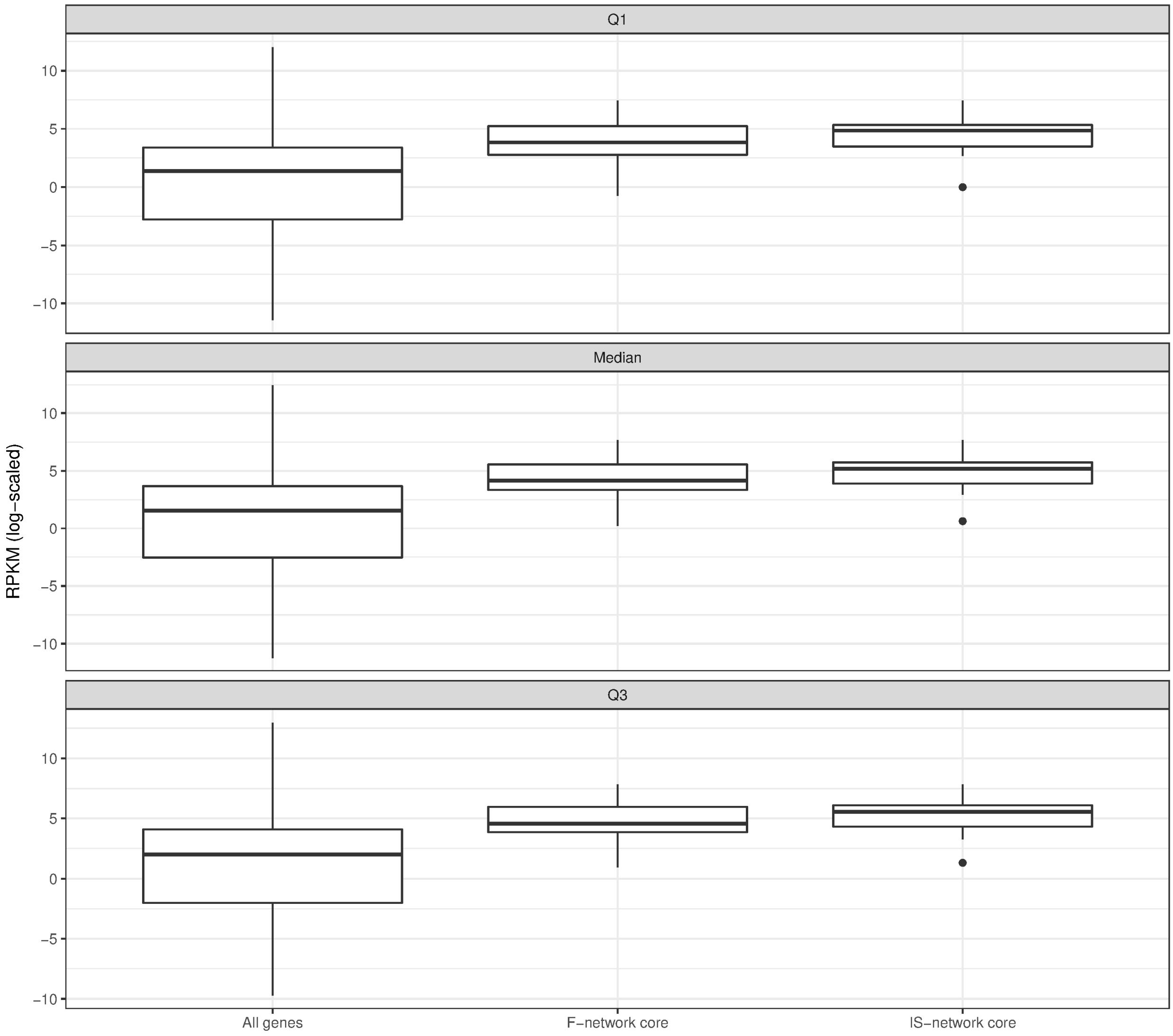

Supplementary Figure 7 | Boxplots for expression levels. Boxplots are shown for the complete set of genes (ca. 26 thousand), for the genes comprising the core of the F-network, and for the genes comprising the core of the IS-network. The data is presented for the lower quartile (Q1), median, and upper quartile (Q3) of expression levels (RPKM) in the collection of cell lines.

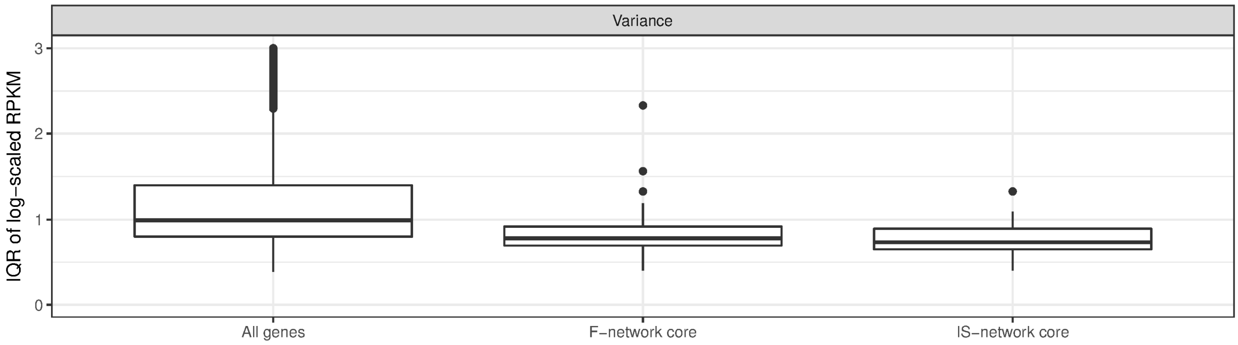

Supplementary Figure 8 | Boxplots for expression variance. Boxplots are shown for the complete set of genes (ca. 26 thousand), for the genes comprising the core of the F-network, and for the genes comprising the core of the IS-network. To increase readability, we set maximum value for the Y axis to three, cutting outliers for the complete set of genes (the outliers have IQR of $\log_2$-scaled RPKM up to 10.6).

Expression levels of core genes in tissue specimens

Analysis of expression levels of core genes in tissue specimens obtained from breast and colorectal cancer patients utilized the microarray series used as training datasets for construction of prognostic gene signatures: GSE17705 for breast cancer patients and GSE39582 for colorectal cancer patients. The series were analyzed separately. Probesets with no associated Gene Symbols were excluded from the analysis. If multiple probesets were associated with the same gene, only the one with the highest median expression level was retained. The Affymetrix Human Genome U133 Plus 2.0 Array that was used for gene expression profiling in GSE39582 series contained probesets for all core genes. The Affymetrix Human Genome U133A Array that was used in GSE17705 series contained probesets for 17 out of 21 genes comprising the core of the F-network, including 10 out of 12 genes comprising the core of the IS-network.

Ranking of genes by median expression level, as well as by the third (higher) or by the first (lower) quartile, demonstrated that core genes have high or at least medium expression levels. All genes comprising the core of the IS-network belonged to the upper half of the ranking (binomial test p-value 9.8×10^{-4} and 2.4×10^{-4} for the breast cancer patients and colorectal cancer patients, respectively). Moreover, in the case of colorectal cancer, 11 out of 12 genes (or even all 12 genes for ranking by the first quartile) belonged to the upper quarter (binomial test p-value 2.2×10^{-6}). In the case of breast cancer, 6 out of 10 IS-core genes with known expression levels belonged to the upper quarter (binomial test p-value 0.020). Similarly, in the core of the F-network, all the genes except one — *PDE4D* for breast cancer and *NR6A1* for colorectal cancer — belonged to the upper half of gene ranking by median expression level, as well as by the first or the third quartile (binomial test p-value 1.4×10^{-4} and 1.0×10^{-5} for breast and colorectal tissue specimens, respectively). Although expression levels for different genes cannot be directly compared in case of microarray expression quantification, these data strongly indicate that the core genes were generally highly expressed in the analyzed tissue specimens.

Analysis of expression variances, quantified as the third quartile to the first quartile ratio of expression levels in linear scale, demonstrated that the core genes tended to have lower variance in comparison to the median values. Note that with respect to ranking, this quantification of expression variance is equivalent to the interquartile range of log-scaled expression levels. To exclude the influence of non-expressed and poorly expressed genes on the analysis results, we focused only on genes from the upper half of genes ranking by the first quartile of expression levels. These genes were ranked by expression variances, and the enrichment of the network cores by the genes from the upper quarter of this ranking was assessed. For the colorectal cancer tissue specimens, the statistical significance of the enrichment was slightly violated (binomial test p-value 0.054 and 0.10 for the core of the IS-network and the core of the F-network, respectively). At the same time, for the breast cancer tissue specimens, the enrichment was statistically significant (binomial test p-value 0.020 and 0.027 for the IS-network and for the F-network, respectively). Moreover, in case of breast cancer, only two and four genes from the core of the IS-network and the F-network, respectively, did not belong to the upper half of the ranking by expression variance.

Datasets utilized for construction of prognostic gene signatures for colorectal cancer patients

Datasets utilized for construction of prognostic gene signatures for colorectal cancer patients are presented in Supplementary Table 6.

Supplementary Table 6 | Datasets utilized for construction of prognostic gene signatures for colorectal cancer patients.

Dataset accession	*Dataset type*	*Patients*		
		Total	*Recurrence within 3 years*	*No recurrence and follow-up of at least 4 years*
GSE39582	Training	519	124	245
GSE37892	Filtration	129	32	48
GSE17536	Filtration	144	31	58
GSE14333	Validation	225	41	85

Prognostic gene triples

The classifiers based on the identified triples of genes from the core of the constructed network had the following parameters.

For the triple *HNRNPK, PANK3, SMC4* weights *w* were –0.142, –0.496 and 1.219, respectively, and the value of ρ was –0.193.

For the triple *MCM7, PANK3, SMC4* weights *w* were 0.061, –0.457 and 1.101, respectively, and the value of ρ was –0.169.

For the triple *MAP2K4, PANK3, SMC4* weights *w* were –0.452, –0.402 and 1.030, respectively, and the value of ρ was –0.141.

For the triple *FASTKD2, PANK1, HUWE1* weights *w* were –0.623, –0.417 and 0.282, respectively, and the value of ρ was –0.044.

For breast cancer, among 680 considered triples of genes from the core of the F-network, 250 passed thresholds on sensitivity and specificity for the training dataset. The first filtration dataset kept 163 triples, and finally after the filtration based on the second filtration dataset 17 triples remained. Out of these 17 triples, four included genes only from the core of the IS-network.

For colorectal cancer, among 1330 considered triples of genes from the core of the F-network, 194 triples satisfied the thresholds for the training dataset, 150 triples survived the first filtration, but only 3 triples survived filtration based on the second filtration dataset (two of these three triples included only genes from the core of the IS-core). Interestingly, filtration based solely on the second filtration set (without filtration based on the first dataset) provided almost the same result, keeping 4 triples.

In spite of relatively low thresholds used for filtration, both for breast cancer and colorectal cancer all triples that survived the filtration confirmed their prognostic potential as for the validation dataset they gave AUC value of at least 0.6.

Interplay of the IS-network core and Myc

The interplay between the genes forming the core of the IS-network and a number of well-studied transcription factors (including p53, Myc family, Fos family, Jun family) was analyzed based on published papers. The sources were found in PubMed and Google Scholar using search requests of the form "*G* and *T*", where *G* and *T* were a gene from the network core and a transcription factor, respectively (or, more precisely, gene symbols associated with the gene and the transcription factor). The analysis showed that in contrast with other studied transcription factors, the Myc family has a remarkable functional connection with the core of the IS-network.

Myc family proteins activate transcription of *MCM7*[2,3] and *RCL1*[4,5] genes and inhibit expression of *CTDSP1*[6] and *ABCF1*[7] genes. For *EIF4H* and *HNRNPK*, binding of N-myc at their promoters has been shown, as well as N-myc-dependent expression of intronic miRNAs of these genes[8]. Furthermore, products of the *EIF4H* and *HNRNPK* genes enhance translation of c-Myc

mRNA[9,10]. For the genes *SMC4, MCM7, FASTKD2,* and *HUWE1,* a direct protein-protein interaction with c-Myc or N-Myc has been reported[11-14].

Additionally, Myc regulates expression of intronic miRNAs that induce intergenic interactions of the core genes represented by edges of the network. Specifically, it activates expression of the mir-15b/16-2 cluster[15], the mir-25/93/106b cluster[3], mir-7-1[16], and mir-590[8], and inhibits expression of mir-26b[6], mir-101[17], and mir-877[8]. Furthermore, miR-16-5p, miR-25-3p and miR-98-5p target c-Myc[18,19], miR-103 targets the c-Myc activators c-Myb and *DVL1*, thereby reducing c-Myc expression[20], and miR-101-3p targets N-Myc[18,19,21].

The described interplay between the core of the IS-network and the Myc family of transcription factors is summarized in Supplementary Fig. 9.

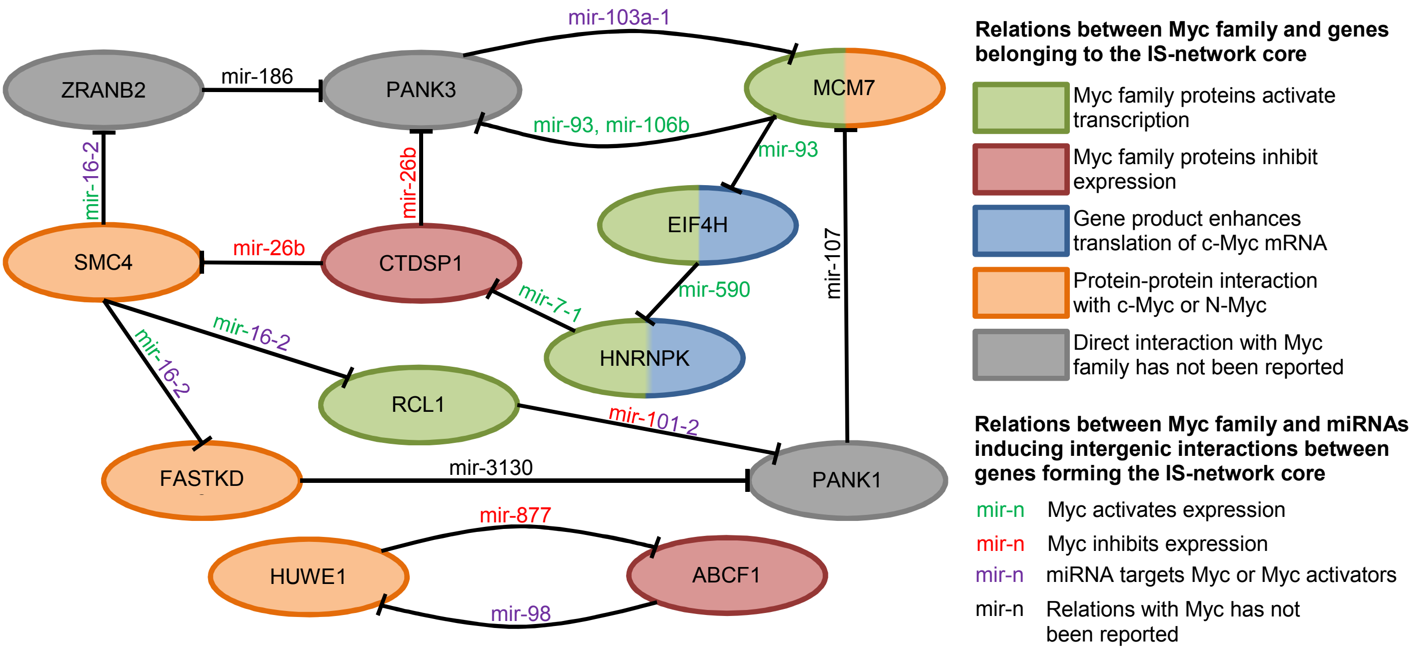

Supplementary Figure 9 | Interplay of the IS-network core and Myc family of transcription factors.

Cross-validation of the identified triples of genes on the training datasets

Results of 3-fold cross-validation of the identified triples of genes on the training datasets are summarized in Supplementary Table 7. For each triple 10,000 cross-validation runs were performed, preserving initial proportion of classes in folds.

In case of 10-fold cross-validation variation of the characteristics was expectedly higher, primarily due to a very small number of patients with recurrence in test fold, which resulted in a very coarse discretization. Yet, the results were highly consistent with the results of 3-fold cross-validation and additionally proved the absence of overfitting. Specifically, lower quartiles of sensitivity and specificity obtained by 10,000 cross-validation runs were at least 50% for all the triples, and lower quartiles for AUC was at least 0.6. Moreover, median AUC was 0.697 for the colorectal cancer triple, 0.7 for two breast cancer triples, and 0.733 for the third breast cancer triple.

Supplementary Table 7 | Results of 3-fold cross-validation on the training dataset. For each characteristic the table presents its median value and interquartile range.

Triple	*Pathology*	*Cross-validation results*		
		Sensitivity	*Specificity*	*AUC*
HNRNPK, PANK3, SMC4	Breast cancer	64.3% [50.0%, 71.4%]	62.3% [56.6%, 67.9%]	0.697 [0.654, 0,739]
MCM7, PANK3, SMC4	Breast cancer	57.1% [50.0%, 71.4%]	64.2% [60.4%, 69.8%]	0.690 [0.644, 0.733]
MAP2K4, PANK3, SMC4	Breast cancer	64.3% [57.1%, 71.4%]	66.0% [62.3%, 69.8%]	0.726 [0.686, 0,768]
FASTKD2, PANK1, HUWE1	Colorectal cancer	61.0% [56.1%, 65.9%]	65.4% [61.7%, 69.1%]	0.693 [0.665, 0.721]

Supplementary references

1. Ewens, W. J. On estimating P values by the Monte Carlo method. *Am. J. Hum. Genet.* **72**, 496–498 (2003).
2. Shohet, J. M. et al. Minichromosome maintenance protein MCM7 is a direct target of the MYCN transcription factor in neuroblastoma. *Cancer Res.* **62,** 1123–1128 (2002).
3. Zhao, Z. N. et al. TSA suppresses miR-106b-93-25 cluster expression through downregulation of MYC and inhibits proliferation and induces apoptosis in human EMC. *PLoS One* **7,** e45133 (2012).
4. Ciribilli, Y., Singh, P., Inga, A. & Borlak, J. c-Myc targeted regulators of cell metabolism in a transgenic mouse model of papillary lung adenocarcinoma. *Oncotarget* **7,** 65514–65539 (2016).
5. Tworkowski, K. A. et al. Adenovirus E1A targets p400 to induce the cellular oncoprotein Myc. *Proc. Natl. Acad. Sci. U S A* **105,** 6103–6108 (2008).
6. Chang, T. C. at al. Widespread microRNA repression by Myc contributes to tumorigenesis. *Nat. Genet.* **40,** 43–50 (2008).
7. Porro, A. et al. Direct and coordinate regulation of ATP-binding cassette transporter genes by Myc factors generates specific transcription signatures that significantly affect the chemoresistance phenotype of cancer cells. *J. Biol. Chem.* **285,** 19532–19543 (2010).
8. Shohet, J. M. et al. A genome-wide search for promoters that respond to increased MYCN reveals both new oncogenic and tumor suppressor microRNAs associated with aggressive neuroblastoma. *Cancer Res.* **71,** 3841–3851 (2011).
9. Vaysse, C. *et al.* Key contribution of eIF4H-mediated translational control in tumor promotion. *Oncotarget* **6,** 39924–39940 (2015).
10. Evans, J. *et al.* Members of the poly (rC) binding protein family stimulate the activity of the c-myc internal ribosome entry segment in vitro and in vivo. *Oncogene* **22,** 8012–8020 (2003).
11. Murakami-Tonami, Y. *et al.* Inactivation of SMC2 shows a synergistic lethal response in MYCN-amplified neuroblastoma cells. *Cell Cycle* **13,** 1115–1131 (2014).
12. Dominguez-Sola, D, *et al.* Non-transcriptional control of DNA replication by c-Myc. *Nature* **448,** 445–451 (2007).
13. Ewing, R. M. *et al.* Large-scale mapping of human protein-protein interactions by mass spectrometry. *Mol. Syst. Biol.* **3,** 89 (2007).
14. Adhikary, S. et al. The ubiquitin ligase HectH9 regulates transcriptional activation by Myc and is essential for tumor cell proliferation. *Cell* **123,** 409–421 (2005).
15. Mestdagh, P. *et al.* MYCN/c-MYC-induced microRNAs repress coding gene networks associated with poor outcome in MYCN/c-MYC-activated tumors. *Oncogene* **29,** 1394–1404 (2010).
16. Chou, Y. T. *et al.* EGFR promotes lung tumorigenesis by activating miR-7 through a Ras/ERK/Myc pathway that targets the Ets2 transcriptional repressor ERF. *Cancer Res.* **70,** 8822–8831 (2010).
17. Wang, L. *et al.* c-Myc-mediated epigenetic silencing of MicroRNA-101 contributes to dysregulation of multiple pathways in hepatocellular carcinoma. *Hepatology* **59,** 1850–1863 (2014).
18. Vlachos, I. S. *et al.* DIANA-TarBase v7.0: indexing more than half a million experimentally supported miRNA:mRNA interactions. *Nucleic Acids Res.* **43 (Database issue),** D153–D159 (2015).
19. Chou, C. H. *et al.* miRTarBase 2016: updates to the experimentally validated miRNA-target interactions database. *Nucleic Acids Res.* **44 (D1),** D239–D247 (2016).
20. Kfir-Erenfeld, S. *et al.* miR-103 inhibits proliferation and sensitizes hemopoietic tumor cells for glucocorticoid-induced apoptosis. *Oncotarget* **8,** 472–489 (2017).
21. Buechner, J. *et al.* Tumour-suppressor microRNAs let-7 and mir-101 target the proto-oncogene MYCN and inhibit cell proliferation in MYCN-amplified neuroblastoma. *Br. J. Cancer* **105,** 296–303 (2011).
